# The WDR11 complex facilitates the tethering of AP-1-derived vesicles

**DOI:** 10.1038/s41467-018-02919-4

**Published:** 2018-02-09

**Authors:** Paloma Navarro Negredo, James R. Edgar, Paul T. Manna, Robin Antrobus, Margaret S. Robinson

**Affiliations:** Cambridge Institute for Medical Research, University of Cambridge, Wellcome Trust/MRC Building, Hills Road, Cambridge, CB2 0XY UK

## Abstract

Vesicluar transport of proteins from endosomes to the *trans*-Golgi network (TGN) is an essential cellular pathway, but much of its machinery is still unknown. A screen for genes involved in endosome-to-TGN trafficking produced two hits, the adaptor protein-1 (AP-1 complex), which facilitates vesicle budding, and WDR11. Here we demonstrate that WDR11 forms a stable complex with two other proteins, which localises to the TGN region and does not appear to be associated with AP-1, suggesting it may act downstream from budding. In a vesicle tethering assay, capture of vesicles by golgin-245 was substantially reduced in WDR11-knockout cells. Moreover, structured illumination microscopy and relocation assays indicate that the WDR11 complex is initially recruited onto vesicles rather than the TGN, where it may in turn recruit the golgin binding partner TBC1D23. We propose that the complex acts together with TBC1D23 to facilitate the golgin-mediated capture of vesicles that were generated using AP-1.

## Abstract (web summary)

Trafficking from endosomes to the trans-Golgi network requires recognition of vesicle tethers during membrane docking. Here, the authors identify a complex localised to AP-1 generated vesicles containing WDR11, C17orf75 and FAM91A, which together with TBC1D23 facilitates vesicle capture on Golgi membranes

## Introduction

Eukaryotic cells are compartmentalised into membrane-bound organelles, each with a distinct protein and lipid composition. Transport of molecules between compartments is initiated by the recruitment of coat proteins onto a ‘donor’ membrane. The coat proteins package selected cargo into a vesicle, which then sheds its coat and moves through the cytoplasm along cytoskeletal tracks towards its destination. Docking of the vesicle onto the ‘acceptor’ membrane makes use of proteins that act as tethers, while fusion requires pairing between SNAREs residing in the two apposing membranes^1,2^.

Although many pathways make use of coats, tethers and SNAREs, the actual machinery is different for each pathway. For instance, clathrin coats, together with cargo-binding adaptor proteins, facilitate endocytosis and trafficking between the *trans*-Golgi network (TGN) and endosomes, while other types of coats act at other membranes^3^. Similarly, different types of golgins tether vesicles to different Golgi subcompartments, with *cis*-Golgi golgins capturing endoplasmic reticulum (ER)-to-Golgi vesicles and *trans*-Golgi golgins capturing endosome-to-Golgi vesicles^4,5^. However, in most cases it is not clear how a vesicle formed using a particular type of coat is then recognised by a particular type of tether. This is especially pertinent for clathrin-coated vesicles (CCVs), because the coat falls off the membrane almost immediately after budding, long before the vesicle has reached its destination^6,7^.

We recently carried out a gene-trap screen on KBM7 cells to identify cellular machinery involved in the sorting of acidic cluster-containing proteins^8^. Acidic clusters are sorting signals found in several proteins that use CCVs to cycle between the TGN and endosomes^9^. The screen produced two robust hits. One was the medium subunit of the AP-1 complex (µ1), the major adaptor for intracellular CCVs (i.e., those that traffic between the TGN and endosomes). The other was a protein called WDR11.

WDR11 is a highly conserved protein but it is still relatively uncharacterised. Proposed functions include tumour suppression^10^, transcriptional regulation^11^, trafficking of endocytosed ricin^2^ and control of viral assembly^12^. WDR11 has been reported to localise to the nucleus^11^, to autophagosomes^13^ and to the TGN;^12^ while proposed binding partners include the transcription factor EMX1^11^, the herpes simplex virus protein ICP0^12^ and a protein of unknown function, C17orf75^13^.

In a subcellular proteomics study from our own laboratory, we proposed an interaction between WDR11, C17orf75 and another uncharacterised protein, FAM91A1, based on their remarkably similar fractionation profiles^14^. In contrast, AP-1 and other CCV components were found to have very different fractionation profiles^14^. However, WDR11 and AP-1 show similar phenotypes upon depletion, both in our acidic cluster sorting screen and in a ricin toxicity screen^13^. Thus, it seems likely that the two proteins contribute to the same pathway rather than physically associate with each other.

In the present study, we have used several approaches to learn more about what WDR11 actually does, and how it fits in with clathrin/AP-1-mediated vesicle budding. We find that WDR11 is part of a stable complex, which appears to be specific for vesicles generated using AP-1, and which contributes to the tethering of these vesicles by interacting indirectly with the TGN-associated golgin, golgin-245, via the recently described ‘bridging factor’ TBC1D23^15^.

## Results

### WDR11 facilitates acidic cluster protein trafficking

**Fig. 1:**
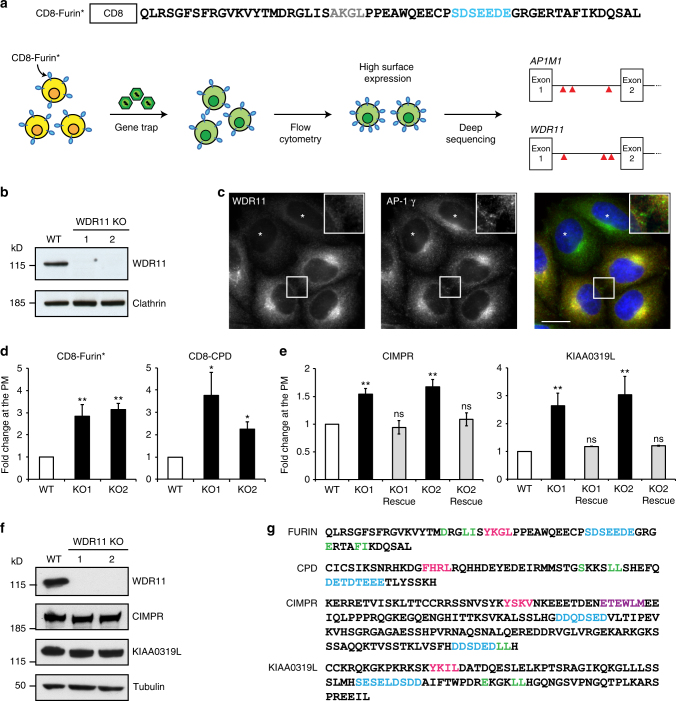
Identification of WDR11 as a protein involved in acidic cluster sorting. **a** A CD8-Furin* chimera was constructed with a mutation in the YKGL sorting signal (AKGL). Blue: acidic cluster. Near-haploid KBM7 cells stably expressing this construct were infected with a gene-trap retrovirus. Mutant cells with increased surface expression of the construct were isolated by flow cytometry, DNA was extracted and viral insertions were identified by deep sequencing. Two hits were found, encoded by the *AP1M1* and *WDR11* genes. **b** The *WDR11* gene was disrupted in HeLa cells and the knockout was confirmed by western blotting, using clathrin heavy chain as a loading control. **c** A mixed population of wild-type and WDR11-knockout cells was double labelled for WDR11 and the AP-1 γ subunit. The insert shows that WDR11 (red) does not display the punctate peripheral labelling seen with anti-γ (green). Scale bar: 20 µm. **d** Wild-type and WDR11-knockout cells were transfected with plasmids encoding CD8-Furin* or CD8-CPD, together with a GFP-encoding plasmid to control for transfection efficiency. Flow cytometry showed that the knockout caused an increase in the surface expression of both constructs. **e** Surface levels of endogenous CIMPR and KIAA0319L were quantified in wild-type cells, WDR11-knockout cells and WDR11-knockout cells transfected with GFP-tagged WDR11. The knockout caused an increase in surface expression of both proteins, which could be rescued with tagged WDR11. In **d** and **e** error bars: S.E.M.; one-way ANOVA and Bonferroni post hoc test; **P ≤ 0.01, *P ≤ 0.05, ns *P* > 0.05; *n* = three independent experiments. **f** Western blots of control and WDR11-knockdown cells, showing that the total amounts of endogenous CIMPR and KIAA0319L are unchanged. **g** Sequences of the cytoplasmic tails of the four proteins, with known or potential sorting signals indicated in colour colour (pink: YXXΦ; green: dileucine; blue: acidic cluster; purple: retromer sorting signal)

Our forward genetic screening approach is shown diagrammatically in Fig. 1a. KBM7 cells were stably transduced to express a chimera containing the extracellular/lumenal and transmembrane domains of CD8 and a cytoplasmic tail derived from furin, which normally localises mainly to the TGN^16,17^. The cells were then infected with a gene-trap retrovirus and sorted by flow cytometry for increased surface expression of the construct. Disrupted genes were identified by deep sequencing. Two robust hits were found, *AP1M1* and *WDR11*, both with three independent insertions into the first intron.

To investigate the phenotype of WDR11 deficiency in a more amenable system, we disrupted the gene in HeLa cells using CRISPR/Cas9 and two different guide RNAs (gRNAs). One clone was selected for each gRNA, and loss of WDR11 was verified by DNA sequencing and western blotting (Fig. 1b). To determine the localisation of WDR11, we mixed together wild-type cells and (as a control) WDR11-knockout cells, then double labelled for WDR11 and the γ subunit of AP-1 (AP-1 γ; Fig. 1c). Endogenous WDR11 was found to have a juxtanuclear pattern, consistent with a TGN localisation, and showed partial overlap with AP-1, at least at the light microscope level. However, WDR11 is more tightly juxtanuclear than AP-1, without the punctate peripheral pattern that has been shown to correspond to endosomes^18–20^ (inset). Moreover, unlike AP-1 and other components of the intracellular clathrin coat, WDR11 is not sensitive to brefeldin A (Supplementary Figure 1). These observations provide further support for the hypothesis that WDR11 contributes to the AP-1 pathway without being physically associated with AP-1.

To confirm that the loss of WDR11 causes an increase in the surface expression of acidic cluster-containing proteins, we transiently transfected wild-type and WDR11-knockout HeLa cells with plasmids encoding either the CD8-Furin* chimera used in our screen, or a CD8 chimera with its tail derived from carboxypeptidase D (CPD), another acidic cluster-containing protein sorted by AP-1^9^. Flow cytometry revealed that there was an ∼3-fold increase in surface expression of both CD8-Furin* and CD8-CPD, in both of the WDR11-knockout cell lines (Fig. 1d), validating the KBM7 cell phenotype. We also found an increase in the surface expression of two endogenous AP-1 cargo proteins with acidic cluster sorting signals, the cation-independent mannose 6-phosphate receptor (CIMPR) and a protein of unknown function, KIAA0319L, even though the overall levels of these two proteins were unchanged (Fig. 1e–g). Moreover, we were able to rescue the phenotype by transfecting the knockout cells with green fluorescent protein (GFP)-tagged WDR11 (Fig. 1e). Together, these experiments demonstrate that in the absence of WDR11, proteins that are normally mainly intracellular become mislocalised and spill over onto the plasma membrane.

### WDR11 forms a stable complex with FAM91A1 and C17orf75

**Fig. 2:**
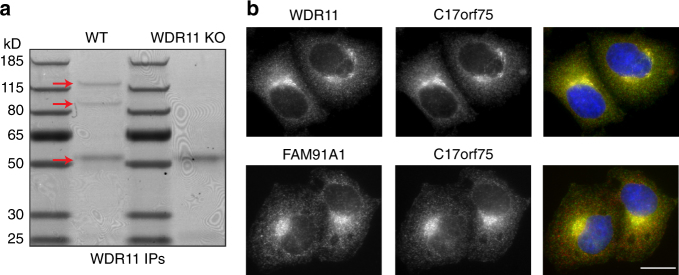
Identification of the WDR11 complex. **a** Extracts from wild-type and WDR11-knockout cells were immunoprecipitated with anti-WDR11, and precipitating proteins were identified by mass spectrometry. The arrows on the Coomassie blue-stained gel indicate, from top to bottom, WDR11, FAM91A1, and C17orf75 (running close to the IgG heavy chain band). **b** HeLa cells were double labelled for either WDR11 (green) and C17orf75 (red), or FAM91A1 (red) and C17orf75 (green). The proteins have virtually identical localisation patterns. Scale bar: 20 µm

To identify binding partners for WDR11, we carried out immunoprecipitations under non-denaturing conditions, using our knockout cells as a control, and identified co-precipitating proteins by mass spectrometry (Fig. 2a, Supplementary Table 1). Two proteins specifically came down in the wild-type cells, FAM91A1 and C17orf75, the same two proteins identified as potential partners by fractionation profiling^14^. Double labelling with rabbit polyclonal antibodies against either WDR11 or FAM91A1, and a mouse monoclonal antibody against C17orf75, demonstrated that the three proteins colocalise in the TGN region (Fig. 2b).

**Fig. 3:**
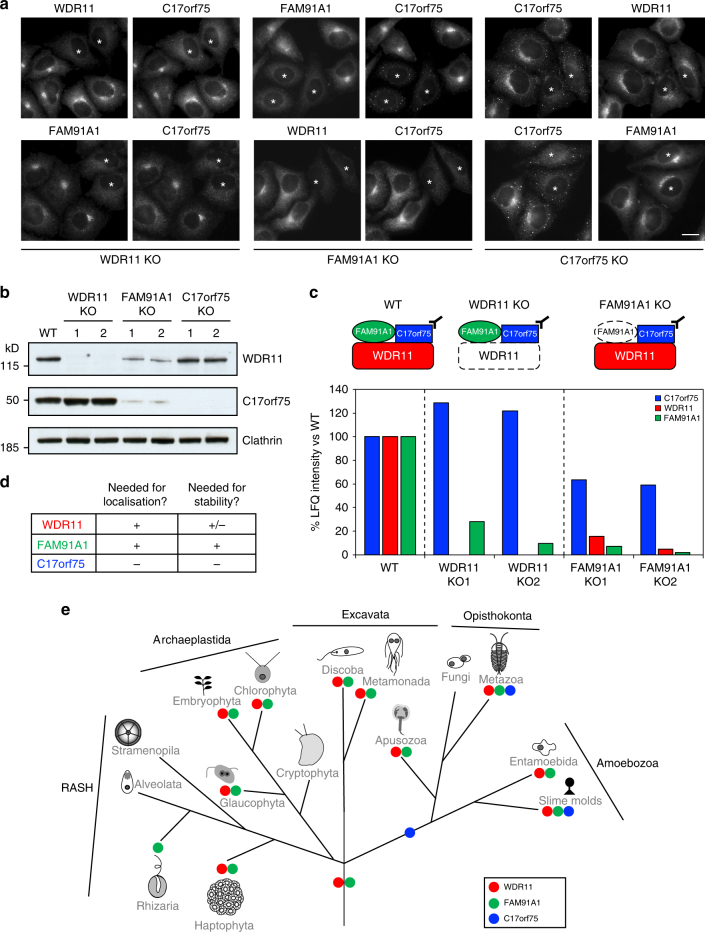
Interdependence of the subunits of the WDR11 complex. **a** The WDR11, FAM91A1 and C17orf75 genes were independently knocked out, then double labelling was used to investigate the localisation of binding partners in mixed populations of wild-type and knockout (asterisks) cells. Scale bar: 20 µm. **b** Western blots of wild-type and knockout cells were probed with antibodies against WDR11, C17orf75 and (as a loading control) clathrin heavy chain. The WDR11 blot is the same one shown in Fig. 1b. **c** C17orf75 and associated proteins were immunoprecipitated from wild-type, WDR11-disrupted and FAM91A1-disrupted cell lysates and analysed by mass spectrometry. In addition to revealing changes in protein stability and/or interactions, the data show that the FAM91A1 disruption was not a true knockout. **d** Summary of the results of **a**, **b** and **c**. **e** Eukaryotic tree of life showing the inferred point of origin of the genes encoding the three subunits, and which proteins are present in various modern organisms

Some protein complexes, such as AP-1, require all of their subunits for stability and function^21,22^, while others contain subunits that are able to function autonomously. To investigate the relationship between WDR11, FAM91A1 and C17orf75, we used CRISPR/Cas9 to knockout either FAM91A1 or C17orf75 in HeLa cells, then validated the gene disruptions by immunofluorescence, DNA sequencing and (in the case of C17orf75) western blotting. Immunofluorescence was then used to investigate the localisation of each of the subunits in cells that had lost one of them. Because of antibody incompatibility, we could not double label for WDR11 and FAM91A1; however, C17orf75 was lost from the TGN region in both WDR11-disrupted and FAM91A1-disrupted cells (Fig. 3a, asterisks), so in both cases we could identify the knockout cells by their loss of juxtanuclear C17orf75. We found that both WDR11 and FAM91A1 are essential for proper localisation of the complex. In contrast, knocking out C17orf75 did not affect the localisation of the other two proteins, indicating that it is more dispensable.

These findings were extended by probing western blots of control and knockout cell homogenates with antibodies against either WDR11 or C17orf75 (the antibody against FAM91A1 is not suitable for western blotting). We found that disrupting WDR11 did not affect the stability of C17orf75, while disrupting FAM91A1 affected the stability of both WDR11 and C17orf75 (Fig. 3b). We also investigated the effect of the WDR11 and FAM91A1 disruptions by performing immunoprecipitations under non-denaturing conditions using the antibody against C17orf75, followed by mass spectrometry (Fig. 3c, d). Consistent with our western blotting results, we found reduced levels of C17orf75 in the immunoprecipitates from the FAM91A1-disrupted cells but not from the WDR11-disrupted cells, as well as reduced levels of WDR11 in immunoprecipitates from the FAM91A1-disrupted cells. We also found less FAM91A1 in the immunoprecipitates from the WDR11-disrupted cells, indicating that it was either less stable or less able to assemble. In addition, we discovered that our disruption of the *FAM91A1* gene, which was verified by sequencing, was not in fact a true knockout, because we could still detect small amounts of FAM91A1 in the immunoprecipitates, which were presumably translated from a different start codon. Therefore, all subsequent experiments investigating knockout phenotypes were carried out on the WDR11-disrupted cells.

Analysis of the phyletic distribution of the three subunits showed that WDR11 and FAM91A1 are present in all five eukaryotic supergroups (Fig. 3e, Supplementary Figure 2). This indicates that both of these proteins were present in the last eukaryotic common ancestor some 1.5 billion years ago. In contrast, C17orf75 was only found in Amoebozoa and Opisthokonta, suggesting that it originated just before these two supergroups diverged. The evolutionary history of the three subunits supports our hypothesis that C17orf75 is more dispensable than WDR11 or FAM91A1.

### AP-1-dependent cargo is missorted in WDR11-knockout cells

**Fig. 4:**
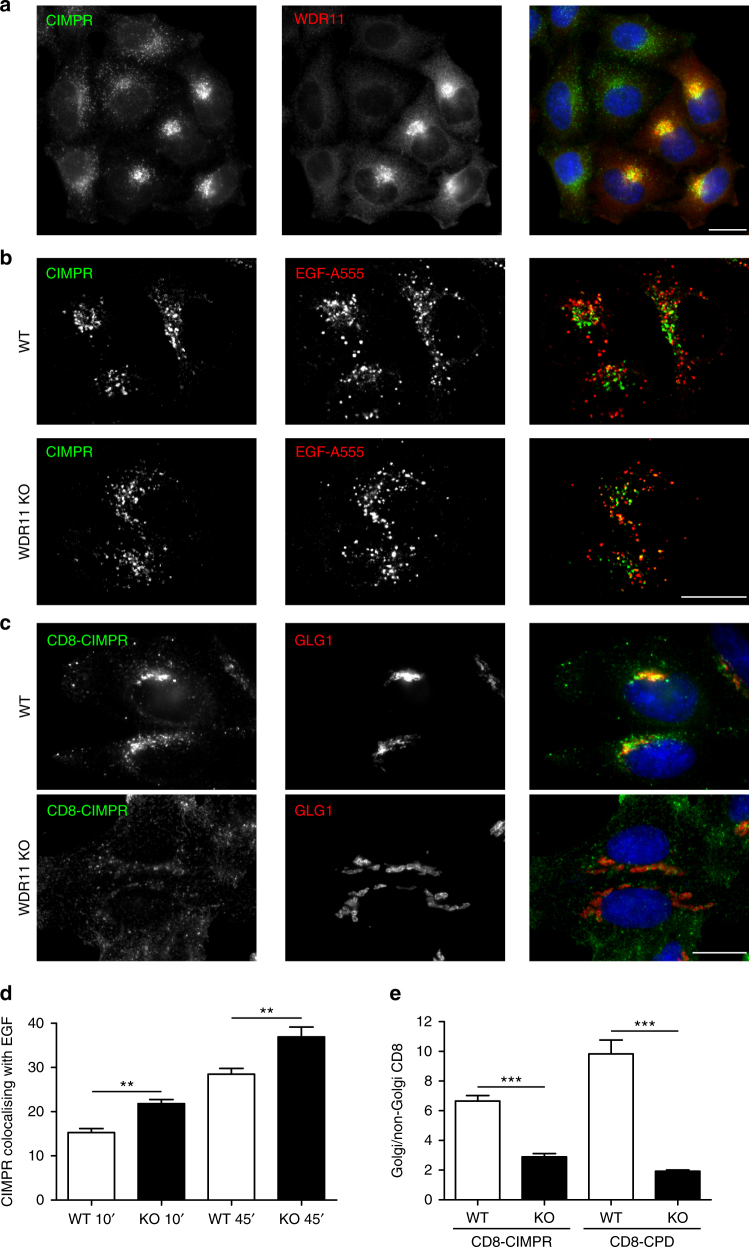
Block in endosome-to-TGN trafficking in WDR11-knockout cells. **a** Widefield image of a mixed population of wild-type and WDR11-knockout cells double labelled for WDR11 and CIMPR. CIMPR has a more peripheral pattern in the knockout cells. **b** Cells were allowed to endocytose fluorescent EGF for 45 min, then double labelled for CIMPR. Representative confocal images show more colocalisation of CIMPR with endocytosed EGF in the knockout cells. **c** Representative widefield images of CD8-CIMPR-expressing cells that were allowed to endocytose anti-CD8 for 15 min, then washed and chased for 45 min. In the wild-type cells, much more antibody reaches the Golgi region (defined by anti-GLG1) than in the knockout cells. Scale bars: 20 µm. **d** Quantification of mean CIMPR-labelling intensity colocalising with internalised EGF (*N* ≥ 20 cells/cell line; three independent experiments). **e** Quantification of the antibody uptake results by automated microscopy in both CD8-CIMPR- and CD8-CPD-expressing cells. Two areas were defined: Golgi (GLG1-positive) and non-Golgi (using a whole cell stain and then subtracting the GLG1-positive region). The ratio of Golgi-to-non-Golgi CD8 fluorescence was calculated for each cell line (≥500 cells were measured per cell line in three independent experiments). Error bars: S.E.M. Data were tested for significance by unpaired two-tailed *t*-tests (**d**) and one-way ANOVA and Bonferroni post hoc test (**e**). ***P ≤ 0.001, **P ≤ 0.01

Loss of WDR11 causes an increase in the levels of AP-1-dependent cargo proteins at the plasma membrane, but is there also a change in their intracellular localisation? Immunofluorescence labelling of a mixed population of WDR11-knockout and wild-type HeLa cells showed that loss of WDR11 causes endogenous CIMPR to move from a mainly juxtanuclear distribution to a more peripheral, punctate distribution (Fig. 4a). There were also changes in the steady-state localisation of CPD and KIAA0319L (Supplementary Figure 3), which could be rescued with exogenous WDR11 (Supplementary Figure 4).

To identify the compartment containing the mislocalised cargo, we allowed wild-type and WDR11-knockout cells to endocytose fluorescent epidermal growth factor (EGF; EGF-A555) for either 10 or 45 min, then labelled the cells with anti-CIMPR. There was substantially more overlap between the two signals in the cells lacking WDR11 at both time points (Fig. 4b, d), suggesting that knocking out WDR11 may cause a defect in endosome-to-TGN trafficking.

To test this hypothesis, we carried out antibody uptake assays. WDR11 was knocked out in HeLa cells stably expressing either CD8-CIMPR or CD8-CPD. The cells were then incubated with anti-CD8 for 15 min at 37 °C, washed, chased for 45 min at 37 °C, fixed and double labelled for endocytosed anti-CD8 and the Golgi-localised glycoprotein GLG1. In wild-type cells, much of the endocytosed antibody localised to the Golgi region, but in WDR11-knockout cells, the antibody accumulated in more peripheral structures (Fig. 4c). To quantify these differences, we defined two areas, Golgi and non-Golgi, and then calculated the Golgi:non-Golgi ratio of mean CD8 fluorescence for each cell line (Supplementary Figure 5). We found a two- to five-fold decrease in Golgi-to-non-Golgi fluorescence in the WDR11-knockout cells (Fig. 4e).

**Fig. 5:**
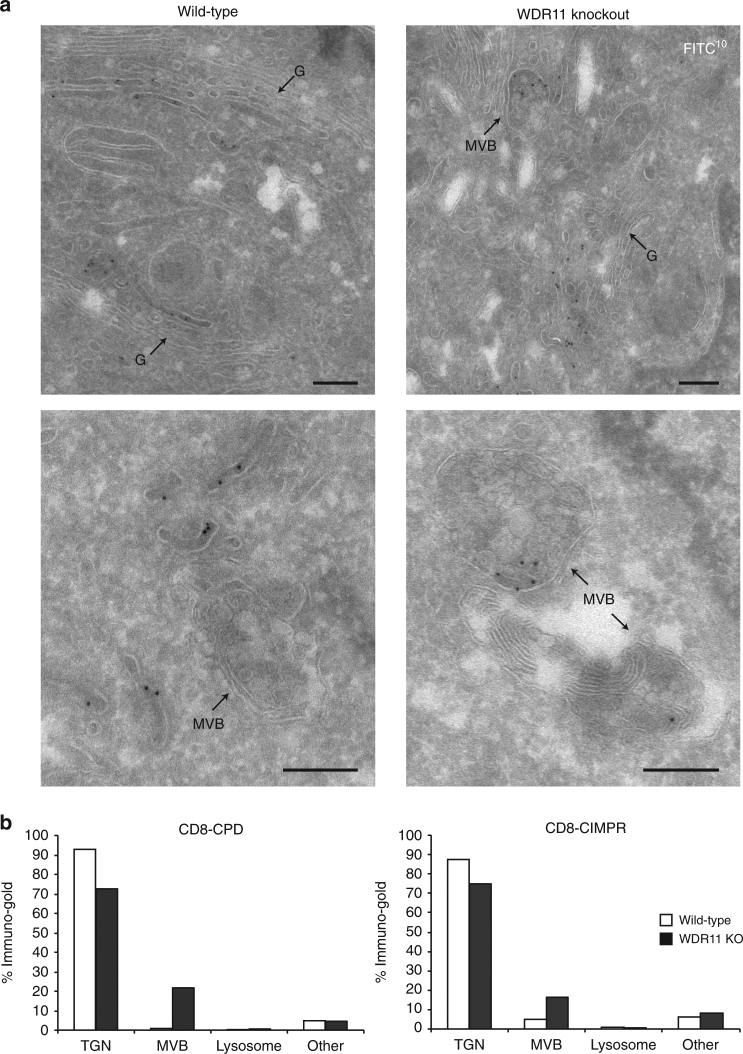
Accumulation of cargo in MVBs in WDR11-knockout cells. **a** CD8-CPD-expressing cells were allowed to endocytose FITC-conjugated anti-CD8 for 3 h, then the endocytosed antibody was localised with anti-FITC using immunogold on cryosections. G: Golgi stacks; MVB: multivesicular bodies. Scale bars: 200 nm. **b** Quantification of EM images from wild-type and WDR11-knockout cells stably expressing either CD8-CPD or CD8-CIMPR. Every gold particle was assigned to an organelle and the percentage labelling per organelle calculated (*N* = 35 cells per cell line). Antibody accumulation in MVBs only occurred in the knockout cells

To visualise the missorting compartment at the electron microscope level, we carried out an antibody uptake assay under somewhat different conditions, in order to maximise labelling efficiency. Cells expressing either CD8-CIMPR of CD8-CPD were allowed to endocytose fluorescein isothiocyanate (FITC)-conjugated anti-CD8 for 3 h at 37 °C, then the endocytosed antibody was localised with anti-FITC followed by protein-A coupled to colloidal gold (Fig. 5a and Supplementary Figure 6). To quantify the labelling, each gold particle was assigned to an organelle. In wild-type cells, very little labelling was observed in multivesicular bodies (MVBs; 4.9% for CD8-CIMPR; 1.1% for CD8-CPD), but there was a dramatic increase in MVB labelling in the WDR11-knockout cells (15.4% for CD8-CIMPR; 21.8% for CD8-CPD), with a concomitant reduction in label associated with tubular and/or TGN compartments (Fig. 5b). This indicates that in the absence of WDR11, the antibody was getting delivered to late endosomes. Together, these observations point to a role for the WDR11 complex in the trafficking of AP-1-dependent cargo proteins from early and/or recycling endosomes to the TGN.

### BioID identifies proteins in proximity to the WDR11 complex

Although the only specific binding partners for WDR11 detected in our immunoprecipitations were FAM91A1 and C17orf75, we suspected that there might be additional interactions that were too transient to be captured by immunoprecipitation. Therefore, we used BioID to try to identify new binding partners. The BioID technique makes use of a promiscuous biotin ligase, BirA*, to biotinylate proteins within a ~10 nm radius^23^. Five cell lines expressing BirA* constructs were generated: one for each of the subunits of the complex; and two control cell lines expressing either BirA* only or BirA*-tagged GFP. Correct localisation of the tagged proteins was confirmed by immunofluorescence (Supplementary Figure 7a). The cells were incubated with biotin for 24 h, then the biotinylated proteins were affinity-purified from cell lysates and identified by mass spectrometry. Relative label-free quantification was performed across four replicate experiments.

**Fig. 6:**
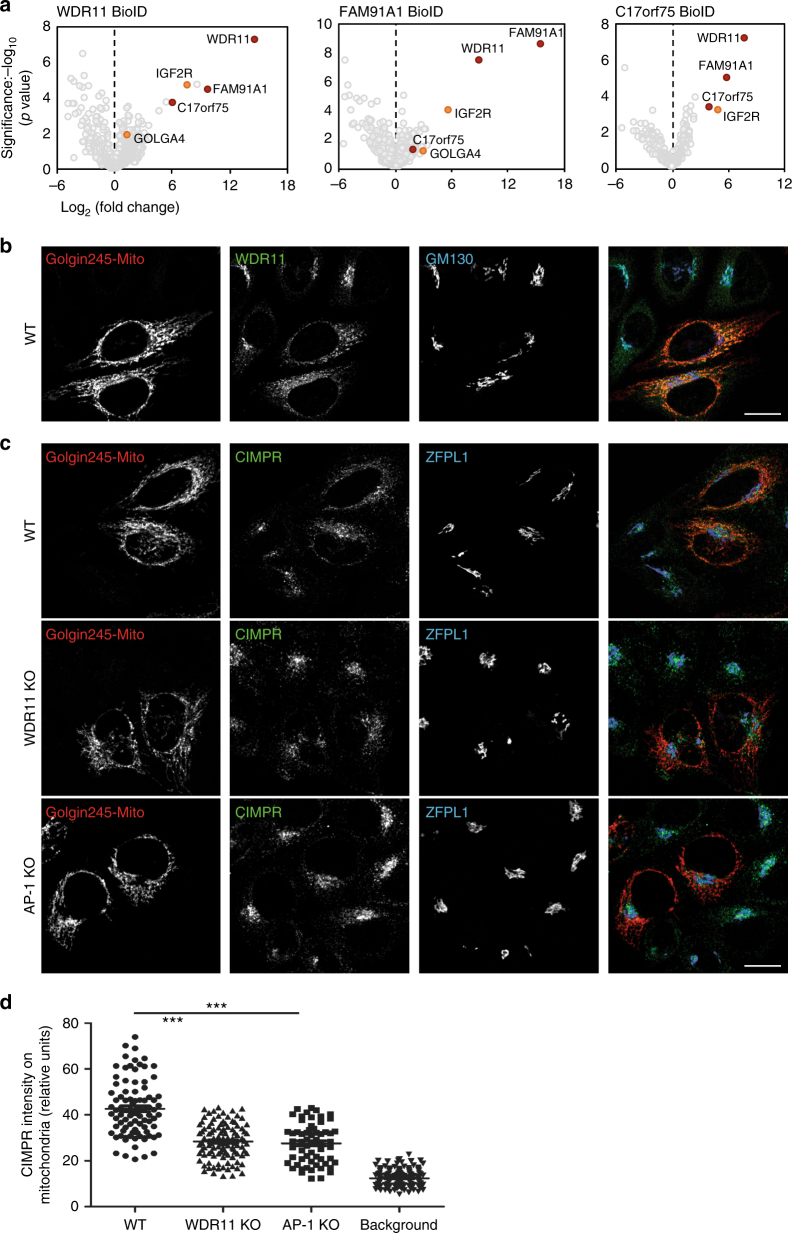
Identification of additional interacting proteins by BioID. **a** BirA* was attached to WDR11, FAM91A1 or C17orf75, and biotinylated proteins were captured with streptavidin. Proteins predicted to be bona fide binding partners are indicated on the volcano plots. IGF2R = CIMPR; GOLGA4 = golgin-245. **b** Golgin-245 was ectopically localised to the mitochondrial outer membrane (golgin-245-mito) and cells were triple labelled for WDR11 and the Golgi protein GM130. Expression of golgin-245-mito causes WDR11 to relocate to mitochondria. **c** Golgin-245 was ectopically localised to the mitochondrial outer membrane (golgin-245-mito) and cells were triple labelled for CIMPR and the Golgi protein ZFPL1. The ability of golgin-245-mito to relocate CIMPR-containing vesicles is markedly reduced in both WDR11- and AP-1-knockout cells. Scale bars: 20 µm. **d** Quantification of mean CIMPR-labelling intensity within the mitochondrial segment (golgin-245) or elsewhere in the cell (background labelling). *N* ≥ 50 cells per cell line; three independent experiments; error bars: S.E.M.; one-way ANOVA and Bonferroni post hoc test; ***P ≤ 0.00

Figure 6a shows volcano plots of the analyses, in which proteins are ranked according to their statistical *P* value and relative abundance in the WDR11, FAM91A1 and C17orf75 BirA* cell lines versus controls. In all three cases, the other subunits of the complex (marked with red circles) were enriched. There was also strong enrichment of the cargo protein CIMPR (*IGF2R*) in all three data sets. Other proteins were enriched in only one of the data sets, and most of these are unlikely to be involved in membrane traffic. However, Rab6A was a hit in the WDR11 data set, and golgin-245 (*GOLGA4*) and TBC1D23 were hits in the FAM91A1 data set (Fig. 6a, Supplementary Figure 7b and Supplementary Table 2). Moreover, Shin et al.^15^ had found both FAM91A1 and TBC1D23 as hits from similar experiments using BirA*-tagged golgin-245 and golgin-97. Because golgins 245 and 97 are tethering proteins that act at the TGN^5^, where they recruit vesicles containing endosome-to-TGN cargo proteins^4^, this suggested a possible role for the WDR11 complex in vesicle tethering^15^.

### The WDR11 complex assists vesicle tethering via golgin-245

To test the physiological relevance of the link with golgin-245, we made use of the assay developed by Wong and Munro, in which golgins are ectopically localised to the mitochondrial outer membrane, where they are still able to capture vesicles. We first investigated the localisation of the WDR11 complex in cells that had been transiently transfected with mitochondrial golgin-245 (golgin-245-mito)^4^. WDR11 was found to be rerouted to mitochondria (Fig. 6b), validating the BioID data. Next, we transfected golgin-245-mito into both wild-type and WDR11-knockout cells, then labelled for endogenous CIMPR (Fig. 6c). In the wild-type cells, much of the CIMPR labelling was found to colocalise with golgin-245-mito, as previously reported by Wong and Munro^4^. However, there was considerably less recruitment of the CIMPR to mitochondria in the WDR11-knockout cells, indicating that the WDR11 complex acts together with golgin-245 to facilitate vesicle capture.

Golgin-245 captures not only CIMPR but also other AP-1-dependent cargo proteins such as CDMPR and Vti1a^4^, suggesting that it might specifically tether AP-1-derived transport vesicles. To test this possibility, we transfected golgin-245-mito into two independent AP-1-knockout clones^8^. The phenotype was virtually identical to that of WDR11-knockout cells (Fig. 6c, d), indicating that AP-1 may be needed to form the vesicles that are then tethered by the WDR11 complex. Neither knockout completely abolished the rerouting of CIMPR-containing vesicles to mitochondria, however, suggesting that some of the vesicles might be generated using other types of machinery, such as retromer and/or sorting nexins.

**Fig. 7:**
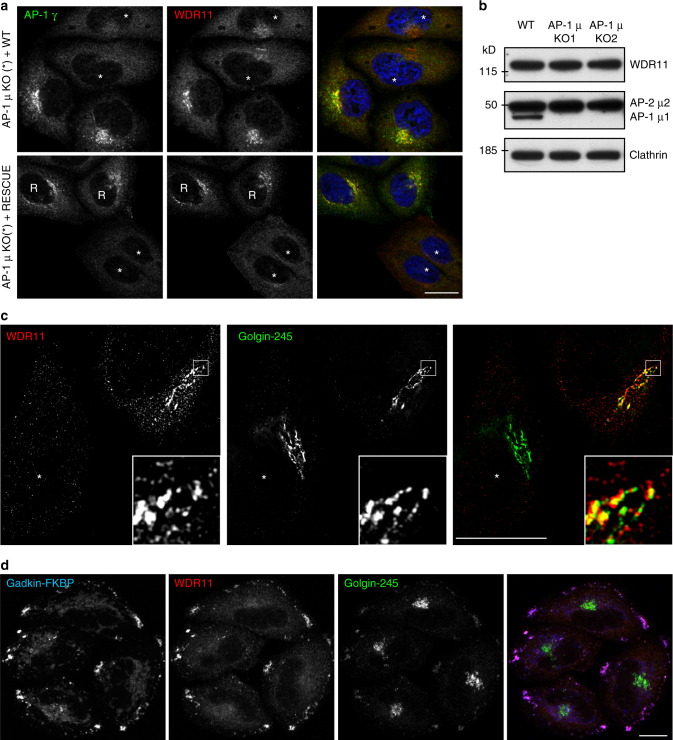
Characterisation of the WDR11-binding compartment. **a** Mixed populations of wild-type and AP-1-knockout cells, or AP-1-knockout cells and rescued AP-1-knockout cells, were stained for WDR11 and AP-1 γ. The juxtanuclear localisation of WDR11 is diminished in AP-1-knockout cells (asterisks), but appears normal in the rescued cells (R). **b** Expression of WDR11 is unaffected in AP-1-knockout cells. **c** Structured illumination microscopy of wild-type and WDR11-knockout cells double labelled for WDR11 and endogenous golgin-245. There is incomplete colocalisation of the two proteins and additional punctate labelling for WDR11. **d** Gadkin-FKBP ‘knocksideways’ cells were treated with rapamycin (200 ng/ml) for 30 min to induce AP-1-derived vesicles to become crosslinked to mitochondria and pulled out to the cell periphery. The cells were then labelled for gadkin-FKBP, WDR11 and golgin-245. WDR11 moves out to the cell periphery, while golgin-245 remains mainly juxtanuclear. Scale bars: 20 µm

To what extent do the WDR11 complex and the AP-1 complex depend upon each other for function? Although we did not see any abnormalities in the distribution of AP-1 in the WDR11-knockout cells (Fig. 1c), we found that knocking out AP-1 caused WDR11 to adopt a much more diffuse distribution (Fig. 7a). The total amount of WDR11 was unchanged, however (Fig. 7b), and subcellular fractionation indicated that much of it was still membrane-bound (Supplementary Figure 8). These observations suggest that without AP-1, the complex can still be recruited, but onto different membranes.

What membranes does the WDR11 complex normally associate with? One possibility is that it may be recruited onto the TGN, by binding (directly or indirectly) to golgin-245, where it lies in wait for incoming AP-1-derived vesicles. Alternatively, it might be recruited initially onto the vesicles. We addressed this question in two ways. First, to localise WDR11 and golgin-245 more precisely, we used super-resolution structured illumination microscopy (SR-SIM). Although the two labels still overlapped, there were distinct differences in the two patterns. In particular, the WDR11 antibody labelled a cloud of dots in the TGN region, which is unlikely to be background because we did not see such a cloud in WDR11-knockout cells (Fig. 7c, asterisk). This suggests that much of the complex is associated with vesicles.

Our second approach was to make use of a cell line we had generated for an earlier ‘knocksideways’ study, which coexpresses FKBP-tagged gadkin (a palmitoylated AP-1-binding partner that also binds kinesin-1 and the Arp2/3 complex) and FRB-tagged Mitotrap (a mitochondrial trapping construct). Addition of rapamycin to these cells causes AP-1-derived vesicles to become crosslinked to mitochondria and pulled out to the cell periphery^24^. Under these conditions, WDR11 was dramatically relocated to the perimeter of the cell, while golgin-245 remained mainly juxtanuclear (Fig. 7d). This observation provides further evidence that WDR11 is initially recruited onto AP-1-derived vesicles, which then become attached to golgin-245 at the TGN.

### The WDR11 complex may act upstream of TBC1D23

Shin et al.^15^ provide compelling evidence that TBC1D23 links the WDR11 complex to golgins, including the demonstration that loss of TBC1D23 causes the complex to adopt a diffuse rather than a Golgi-localised distribution. Although at first this finding seems to suggest that TBC1D23 recruits the WDR11 complex onto membranes, an alternative explanation is that the WDR11 complex is still attached to membranes in the absence of TBC1D23, but those membranes are difficult to distinguish because they are in the form of small vesicles spread throughout the cell rather than concentrated in a particular region. The observation that WDR11-positive vesicles cluster at the cell periphery in gadkin knocksideways cells enabled us to carry out further investigations into the sequence of events.

**Fig. 8:**
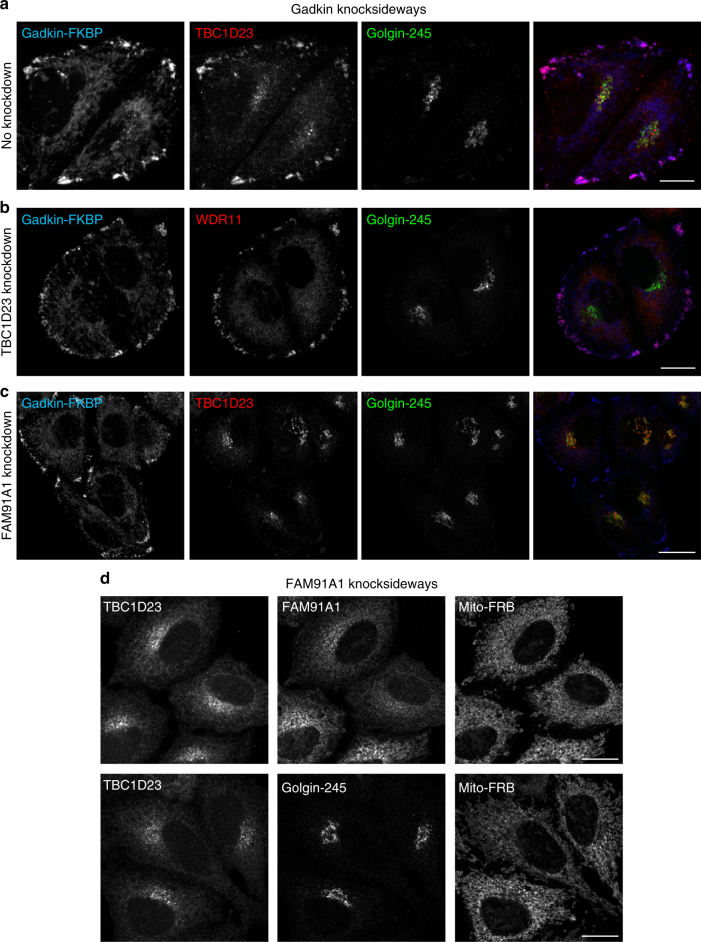
Recruitment of TBC1D23 by the WDR11 complex. **a** Gadkin knocksideways cells were treated as in Fig. 7d and then labelled for gadkin-FKBP, TBC1D23 and golgin-245. Like WDR11, much of the TBC1D23 is relocated to the cell periphery. **b** Knocking down TBC1D23 does not affect the ability of the WDR11 complex to be redistributed in gadkin knocksideways cells. **c** In contrast, knocking down FAM91A1 prevents TBC1D23 from being redistributed in gadkin knocksideways cells, causing it to remain colocalised with golgin-245. **d** FAM91A1 knocksideways causes TBC1D23 to be relocated to mitochondria, while the localisation of golgin-245 does not change. Scale bars: 20 µm. Representative confocal images are shown from three independent experiments

First, we localised TBC1D23 in the gadkin knocksideways cells, and found that much of it was relocated to the cell periphery, where it colocalised with gadkin-FKBP (Fig. 8a). Next, we examined the effect of knocking down either TBC1D23 or a component of the WDR11 complex. Depleting TBC1D23 did not affect the colocalisation of the WDR11 complex with gadkin-FKBP (Fig. 8b). However, when we depleted components of the WDR11 complex, TBC1D23 no longer moved to the cell periphery in gadkin knocksideways cells, but stayed in the Golgi region (Fig. 8c and Supplementary Figure 9a–c). These findings suggest that the WDR11 complex may be recruiting TBC1D23 rather than the other way around.

We also made use of the knocksideways system to relocate the WDR11 complex to mitochondria, by putting an FKBP domain onto FAM91A1 and coexpressing it with Mitotrap. Addition of rapamycin for 10 min not only caused the complex to be rerouted onto mitochondria but also changed the localisation of TBC1D23, while golgin-245 remained in its usual position (Fig. 8d and Supplementary Figure 9d). Thus, at least under these conditions, the WDR11 complex is capable of recruiting TBC1D23. Together, our findings suggest that the WDR11 complex acts upstream of TBC1D23, recruiting it onto AP-1-derived vesicles, which then use TBC1D23 for the next step, docking onto the TGN by interacting with golgins.

## Discussion

In our gene-trap screen for machinery involved in the sorting of acidic cluster-containing cargo, we identified two hits: the µ subunit of AP-1 and WDR11. In the present study, we show that WDR11 facilitates vesicle docking. Thus, our screen turned up machinery acting at both ends of the endosome-to-TGN pathway: the initial vesicle budding stage and the final vesicle tethering stage.

Previous studies on WDR11 suggested it might play some sort of role in membrane traffic, but there was no consensus as to its localisation or function. By comparing wild-type and knockout cells, we were able to show that WDR11 localises to the TGN region and surrounding puncta, and that it facilitates endosome-to-TGN trafficking. Paradoxically, a screen for genes involved in ricin sensitivity showed that silencing WDR11 with short hairpin RNA increased the susceptibility of cells to the toxin^13^. Ricin is trafficked from endosomes back to the ER via the TGN, so one might have expected the cells to become less sensitive to ricin, not more. However, the same study showed that knocking down other machinery involved in retrograde traffic, such as AP-1, retromer and COPI, also sensitised the cells to ricin. It is possible that some of these effects were indirect, caused by upregulation of compensatory pathways and/or the creation of bottlenecks that caused the toxin to take a different route.

Indirect effects may also explain why, in the present study, endosome-to-TGN cargo proteins accumulated on the plasma membrane and in late endosomes rather than in vesicles. A similar phenotype has been seen in yeast cells with deletions in genes encoding subunits of the GARP complex, which also acts as a tethering factor for endosome-to-TGN vesicles: cargo proteins were mislocalised to the vacuole^25^. Clearly the cell needs to maintain homoestasis between vesicle budding and vesicle consumption, and it may do so by somehow allowing vesicles to fuse with the wrong compartment if necessary, rather than to build up indefinitely.

WDR11 is stably associated with two other poorly characterised proteins, FAM91A1 and C17orf75. Secondary structure predictions indicate that all three proteins are mainly folded, and WDR11 is predicted to consist of two seven-bladed β-propellers followed by an α-solenoid (http://www.compbio.dundee.ac.uk/jpred/; https://toolkit.tuebingen.mpg.de/hhpred). This type of architecture is also seen in several of the subunits of the HOPS/CORVET tethering complex^26^, as well as in vesicle coats, nuclear pore components and subunits of the intraflagellar transport complex^27^. Structural studies will be essential for resolving how the complex is put together and how it interacts with other proteins involved in tethering, in particular with TBC1D23, which links the WDR11 complex to golgins^15^.

When does the WDR11 complex get recruited onto the membrane? The complex does not appear to be associated with CCVs (Fig. 1c, Supplementary Figure 1 and Borner et al.^14^), so we propose that it gets recruited onto vesicles some time after budding and uncoating. Evidence that these vesicles were made using AP-1 comes from the finding that all of the cargo proteins that have been identified by biotinylation, relocation to mitochondria and/or mislocalisation are also cargo proteins for AP-1^9^. We think that recruitment of the complex occurs before the vesicles actually reach the TGN, because of our SIM microscopy and gadkin knocksideways experiments (Fig. 7c, d). The gadkin knocksideways experiments also indicate that TBC1D23 is recruited onto the vesicles by the WDR11 complex, rather than the other way around (Fig. 8). Once the vesicles arrive at the TGN, TBC1D23 links them to golgin-245^15^. In the absence of WDR11, vesicles containing AP-1-dependent cargo are no longer able to be guided efficiently to the TGN, and the cargo proteins (including those containing acidic clusters) become mislocalised to other membranes, in particular the plasma membrane and late endosomes.

Even in the absence of WDR11, some CIMPR-containing vesicles can still be captured by golgin-245, although the levels are reduced (Fig. 6). Similarly, Shin et al.^15^ show that there is still cargo capture in FAM91A1-deficient cells, but not in TBC1D23-deficient cells. We suspect that this is because there are distinct populations of endosome-to-TGN carriers, made with different types of machinery, including retromer and sorting nexins as well as AP-1. More than one of these population may make use of TBC1D23. This possibility is supported by the demonstration by Shin et al.^15^ that TBC1D23 binds to the WASH complex, which is recruited onto membranes by retromer^28,29^.

Perhaps the most fundamental question posed by the present study is how the AP-1-derived vesicles are recognised by the WDR11 complex. In some cases of vesicle budding followed by tethering, there is a direct interaction between coats and tethers: for instance, the Sec23 subunit of the COPII coat binds to the Bet3 subunit of the TRAPPI complex^30^. However, this does not appear to be the case for AP-1, which is rapidly removed after the vesicle buds^7^. A more likely scenario is one where the WDR11-containing complex recognises a component of the vesicle that was first put into place by AP-1, such as a rab or a SNARE. Candidates for such components can be found in the list of proteins associated with the AP-1-dependent population of vesicles^9^, as well as in the list of proteins affected by gadkin knocksideways^24^. There is a great deal of overlap between the two lists, but importantly, the WDR11 complex and TBC1D23 appear as hits in the second list only.

However, recruitment of the WDR11 complex onto membranes is not necessarily mediated by a single docking site. A common theme that has emerged in membrane traffic is coincidence detection, where multiple low-affinity interactions help to ensure that a protein gets to the right place at the right time. For instance, recruitment of AP-1 and other intracellular adaptors onto the appropriate membrane requires small GTPases, phosphoinositides, cargo proteins and other coat components. Conformational changes and reversible modifications also contribute to membrane association^3^. It seems likely that similar mechanisms may be involved in the recruitment of tethering proteins. Our discovery of a functional link between AP-1 and the WDR11 complex should help to uncover the mechanisms used by different populations of vesicles to find their targets.

## Materials and methods

### Antibodies

Antibodies used in this study include the following: AP-1 γ (1:1000 for IF and 1:10 000 for WB, mAb100.3, mouse; Sigma-Aldrich); AP-1/2µ (1:100, AP50, mouse, BD Biosciences); C17orf75 (1:200 for IF and 1:500 for WB, ab56381, mouse, Abcam); CD8 (1:500, ATCC number: CRL-8014, mouse, LGC Promochem); CIMPR (for immunofluorescence (IF) 1:300, 2G11, mouse, Abcam; for western blotting (WB) 1:10 000, 1000.3, rabbit, gift from Paul Luzio (University of Cambridge)); clathrin heavy chain (1:10 000, rabbit, made in-house^31^); CPD (1:500, AE142, rabbit, gift from Lloyd Fricker (Albert Einstein College of Medicine)); FAM91A1 (1:200, ab81618, rabbit, Abcam); fluorescein/Oregon Green (1:200, A889, rabbit, Molecular Probes); GLG1 (1:200, AP9839b, rabbit, Abgent); GM130 (1:200, 610822, mouse; BD Biosciences); golgin-245 (1:300, 611281, mouse, BD Biosciences); HA (1:300, 3F10, rat, Roche); KIAA0319L (1:300, ab105385, mouse, Abcam); α-tubulin (1:10 000, DM1A, mouse, Sigma-Aldrich); WDR11 (1:500 for WB and 1:300 for IF, ab93871, rabbit, Abcam); and ZFPL1 (1:800, HPA014909, rabbit, Sigma-Aldrich). Conjugated antibodies used in this study were FITC-labelled anti-CD8 (1:50, 154-040, mouse, Ancell) and Alexa^647^-labelled anti-CD8 (1:50, MCA1226A647, mouse, Serotec). Horseradish peroxidase-labelled secondary antibodies were purchased from Sigma-Aldrich (1:5000). Fluorescently labelled secondary antibodies used in this study were Alexa^488^-labelled donkey anti-mouse IgG (A21202), Alexa^594^-labelled donkey anti-rabbit IgG (A21207) and Alexa^568^-labelled goat anti-rat IgG (A11077), all purchased from Invitrogen and used at 1:500.

### Constructs

The CD8-Furin* construct used for the screen and subsequent validation have been reported by Navarro Negredo et al.^8^). The CD8-CIMPR and CD8-CPD constructs were made by Kouki Harasaki^32^. WDR11 and C17orf75 cDNAs were obtained from Dharmacon (catalogue numbers MHS6278-202806240 and MHS6278-202827852, respectively). The FAM91A1 cDNA and golgin-245ΔCterm-HA-MAO construct were a gift from Sean Munro (MRC LMB). A modified pLXIN retroviral vector encoding BirA* was a gift from Folma Buss (CIMR). Gibson assembly was used to introduce each of the WDR11 complex subunits into the BirA-pLXIN vector. The GFP-tagged WDR11 rescue construct was made by cloning WDR11 cDNA into an eGFP-N2 vector (Clontech) using *Acc*65I/*Bsr*GI and *Not*I restriction sites. The WDR11 rescue construct for stable expression was made by introducing a myc tag at the WDR11 cDNA N terminus by PCR, followed by cloning into a retroviral pQCXIH vector (Clontech) using *Bam*HI and *Not*I sites.

### Cell culture

Cells were cultured at 37 °C and 5% CO_2_. HeLa M cells and HEK 293ET cells were a gift from Paul Lehner (University of Cambridge) and were cultured in RPMI 1640 (Sigma-Aldrich). Retinal pigment epithelial (RPE) cells were a gift from Folma Buss (University of Cambridge) and cultured in 50/50 F12 Ham/DMEM (Sigma-Aldrich). In both cases the medium was supplemented with 10% foetal calf serum (v/v), 2 mM l-glutamine, 100 U/ml penicillin and 100 μg/ml streptomycin (all from Sigma-Aldrich).

Transient DNA transfections were carried out using *Trans*IT-HeLaMONSTER^®^ kit (Mirus, Cambridge Bioscience Ltd, UK) following the manufacturer’s instructions. For stable cell line generation, HEK 293ET cells were co-transfected with the appropriate retroviral vector and the packaging plasmids pMD.GagPol and pMD.VSVG (both a gift from Paul Lehner, University of Cambridge) in a ratio of 50:30:15. The virus-containing supernatant was filtered through a 0.45 µm filter, supplemented with 10 µg/ml hexadimethrine bromide (Polybrene, Sigma-Aldrich) and applied directly to the target cells.

No cell lines used in this study were found in the database of commonly misidentified cell lines that is maintained by ICLAC and NCBI Biosample. The cell lines tested negative for mycoplasma contamination and were regularly treated with mycoplasma removing agent (093050044, MP Biomedicals).

### CRISPR/Cas9-mediated gene disruption

The Zhang online CRISPR design tool (http://crispr.mit.edu/33) was used to identify suitable gRNA targets. The selected gRNAs targeted exon 1 of WDR11, exon 3 of C17orf75 and introns 1 and 2 of FAM91A1 (two gRNAs used together to excise FAM91A1 exon 1)^33^. Each gRNA was ordered as a pair of complementary oligonucleotides (Sigma-Aldrich) with the sequences 5′-CACCGN20-3′ and 5′-AAACN20C-3′, annealed and cloned into into the *Bbs*I site of the dual Cas9 and gRNA expression vector pX330 (Addgene). HeLa M cells were transfected with each pX330 plasmid and pIRESpuro (Clontech) in a ratio of 3:1. The cells were enriched by 2 days of puromycin (1 µg/ml) selection and single-cell clones were isolated by serial dilution. Knockout efficiency was tested by western blotting, immunofluorescence and Sanger sequencing^8^.

The sequencing also identified the nature of the genomic disruptions found in each of the knockout cell lines. For the WDR11 KO1 line, each of the two alleles had a 1 bp deletion, while the KO2 line had a 1 bp deletion in one allele and a 3 bp deletion in the other allele. Three different mutant alleles were found in the FAM91A1 KO1 line: a 144-nucleotide (nt) deletion, a 142-nt insertion, and a 107-nt and 3-nt deletion. In the FAM91A1 KO2 line, only two disruptions were identified, of 107 nt and 108 nt. The C17orf75 knockout line had two mutant alleles, with a 1 and a 3 bp insertion.

### siRNA-mediated knockdown

ON-TARGETplus Human FAM91A1 (157769) set of four siRNAs and TBC1D23 (55773) siRNA SMARTpool were purchased from Dharmacon GE. The target sequences were as follows: CGCAAUCAGUUACGAUAUA, CAAGAAGCUUCAUCGGCAA, GGUAAAUGCAGUAACGGUU and AGAAAUGCGCUGUUGAUAA of Hs FAM91A1; and AGAGAUCCUUCAAGCGAAU, GGGAGAUUGUUUCACGGAA, GCGCUGAAUUCUGUAGUUA and CCGUUAAUGUCAGGGAAAA of Hs TBC1D23.

Gadkin knocksideways cells were cultured to 30–50% confluency before transfection with siRNAs (160 mM) using Oligofectamine (Invitrogen) in Opti-MEM (Sigma-Aldrich) following the manufacturer’s instructions. Two consecutive transfections separated by 48 h were performed. Forty-eight hours after the second transfection the cells were treated with rapamycin for 30 min and analysed by immunofluorescence or harvested for immunoblotting to assess the knockdown efficiency. Three independent experiments were performed.

### FAM91A1 knocksideways

To rapidly reroute FAM91A1 onto the mitochondrial outer membrane myc-FKBP was fused to the C terminus of FAM91A1 cDNA and cloned into the PLXIN retroviral vector by Gibson assembly. The construct was expressed in cells stably expressing Mitotrap-YFP-HA-FRB, a gift from Jenny Hirst (CIMR). Endogenous FAM91A1 was knocked out in these cells using the same CRISPR/Cas9 strategy as before and FAM91A1 knockout was confirmed by genomic DNA sequencing.

### Flow cytometry

Typically, ~10^6^ cells were trypsinised, washed with phosphate-buffered saline (PBS), incubated with primary antibody for 30 min at 4 °C, washed three times with PBS and incubated with fluorophore-conjugated secondary antibody for 30 min at 4 °C. A further three washes in PBS were performed before analysis on a FACSCalibur instrument (BD Biosciences). Data analysis was carried out using CELLQuest (BD Biosciences) and FlowJo v10 software (FLOWJO, LLC). In all, 10 000 live cells (identified by their FSC/SSC profile) were analysed for each sample. CD8 chimeras were co-transfected with an EGFP-N2 plasmid. In these and WDR11-GFP rescue experiments, the GFP fluorescence was used to gate for transfected cells (Supplementary Figure 10). The mean fluorescence intensity was obtained as the geometric mean of the recorded/gated events and the fold change in the surface levels of the labelled protein calculated by dividing the geometric mean for each knockout or rescue condition by that for wild-type cells. The means of at least three independent experiments were pooled for statistical analysis.

### Fluorescence microscopy

For immunofluorescence labelling, cells were plated onto 13 mm glass coverslips, fixed in 3% paraformaldehyde (PFA) and permeabilised with 0.1% Triton-X 100 made up in PBS-bovine serum albumin (BSA; 0.5% BSA in PBS). Primary and fluorophore-conjugated secondary antibodies were diluted in PBS-BSA and incubated for 45 min. Each incubation was followed by at least five washes in PBS-BSA and a final three washes in PBS only. The coverslips were then mounted with ProLong Gold Antifade Reagent (Thermo Fisher Scientific). Widefield fluorescent images were captured with an Axio Imager II microscope (×63/1.4 numerical aperture (NA) oil immersion objective; AxioCam 506 camera; ZEISS), and confocal images were captured with a LSM880 confocal microscope (×63 NA 1.40 oil immersion objective; ZEISS) both equipped with ZEN software (ZEISS). Where representative images are shown, the experiment was repeated at least three times and in each case 40 fields were imaged using the ×63 objective. Where knockout cell lines were used, at least two independent clones generated with different gRNAs were analysed in every case.

Where indicated the cells were treated with 50 µg/ml brefeldin A (B7651, Sigma-Aldrich) for 5, 10 or 30 min or 200 ng/ml rapamycin (Sigma-Aldrich) for 30 min. In both cases the drugs were diluted in media pre-warmed to 37 °C. For the EGF feeding assays, cells were starved in serum-free medium at 37 °C for 90 min and were then incubated with a 1:250 dilution of EGF conjugated to Alexa555 fluorophore (1:250 in media warmed to 37 °C, Thermo Fisher Scientific) for 10, 45 or 90 min at 37 °C. Quantification of the steady-state CIMPR and endocytosed EGF-555 colocalisation was performed with Imaris 8.4 software (Bitpane). The EGF and Golgi regions were segmented by the EGF-555- and ZPL1-staining patterns, respectively. Next, the Golgi segment was subtracted from the EGF segment. The mean CIMPR fluorescence intensity was then determined in the latter segment. Over a hundred cells were analysed for each cell line and time point over three independent experiments. Knockout data are derived from two independent knockout clones.

For SR-SIM, wild-type and WDR11-knockout cells were seeded onto high-precision glass coverslips (Carl Zeiss), processed for conventional immunofluorescence and mounted with ProLong Diamond antifade medium without DAPI (Thermo Fisher Scientific). Images were acquired with an ElyraPS1 super-resolution microscope (Carl Zeiss) using a ×63 1.4 NA plan-apo Carl Zeiss objective lens and Immersol 518F (23 °C) immersion oil. Data sets were collected with five grating phases, five rotations and sufficient z positions spaced 110 nm apart to form an ~2 μm deep volume of raw SR-SIM data. Structured illumination post-processing was performed in ZEN using parameters determined by automated analysis of the data sets. The reconstructed images were then corrected for spherical and chromatic aberrations using channel alignment information. This information was created by imaging, with the same instrument settings, a sample where one primary antibody was simultaneously labelled with two secondary antibodies conjugated to different fluorophores.

### Antibody feeding assays

For immunofluorescence experiments, control and WDR11-knockout (two independent clones) cells stably expressing CD8-CIMPR or CD8-CPD were seeded onto glass-bottomed 96-well plates (3 wells per cell type). The cells were allowed to bind mouse anti-CD8 antibody (1:500) at 37 °C for 15 min, washed and then chased for 45 min at 37 °C before fixing and immunolabelling with rabbit anti-GLG1 (1:200) and secondary antibodies (anti-mouse Alexa 488 and anti-rabbit Alexa 549, 1:500). Whole cell stain blue (Cellomics; 1:200) was used to label whole cells. For quantification, a CellInsight CX7 High Content Screening (Thermo Fisher Scientific) widefield automated microscope was used together with the Cellomics vHCS:View software and the general colocalisation measuring tool. Two areas were selected: Golgi region (GLG1 staining) and non-Golgi region (whole cell minus GLG1 staining), and the average CD8 fluorescence intensity was measured in each region. Three wells in which cells were treated the same but without primary antibodies were used to determine background fluorescence signal, which was subtracted from all measurements. At least 500 cells were analysed for each cell line in three biological repeats. The ratio of CD8 fluorescence in the Golgi versus the non-Golgi region was calculated for each cell line and the means for the two independent knockout clones were pulled together as they were very similar.

For electron microscopy, wild-type and WDR11-knockout HeLa cells stably expressing either CD8-CIMPR or CD8-CPD were incubated in media warmed to 37 °C containing FITC-labelled anti-CD8 (1:50, Ancell) for 3 h at 37 °C.

### Electron microscopy

Cells were fixed with freshly prepared 4% PFA/0.2% gluteraldehyde in 0.25 M HEPES buffer, pH 7.4, for 2 h before being scraped, pelleted and embedded in 12% gelatin. Pellets were infused with 1.7 M sucrose/15% polyvinyl pyrolidone overnight at 4 °C. Ultrathin frozen sections were cut with a diamond knife in a Reichert Ultracut S ultramicrotome (Leica, Germany), collected with methyl cellulose:sucrose and mounted on Formvar-coated electron microscopy grids. Ultrathin sections were labelled with an anti-fluorescein/Oregon Green primary antibody (Molecular Probes) and detected using protein-A conjugated to 10 nm colloidal gold (Utrecht University, The Netherlands). Sections were viewed on a FEI Tecnai transmission electron microscope (Eindhoven, The Netherlands) at 80 kV. Analyses of variance (ANOVAs) followed by Bonferroni post hoc tests were performed to determine whether each knockout clone was statistically different from wild-type cells.

To quantify the extent of labelling, 35 cells were analysed for each condition. Because the cells had been fixed, scraped and pelleted, the organelles within the cryosections were randomly distributed. Every gold particle was counted and assigned to an organelle, based on morphology as either TGN/tubular recycling compartments (compartments adjacent to Golgi stacks, or tubular endosomes), MVBs (endosomal compartments containing intralumenal vesicles but lacking membrane whorls), lysosomes (compartments containing membrane whorls) or other (cytosolic, nuclear, mitochondria and plasma membrane). The percentage of labelling per organelle was calculated for each cell line.

### Golgin mitochondrial recruitment assay

This assay was originally devised by Wong and Munro^4^. Wild-type cells and two independent WDR11-knockout and AP-1-knockout clones were transiently transfected with a golgin-245ΔCterm-HA-MAO construct (a kind gift from Sean Munro), which localises to the outer membrane of mitochondria. Forty-eight hours post transfection the cells were fixed and stained with antibodies against the HA tag (golgin on mitochondria), CIMPR and ZFLP1 (Golgi). Quantification of confocal images was preformed using Imaris 8.4 (Bitplane). The mitochondria and Golgi regions of each transfected cell were segmented by the HA- and ZPL1-staining patters, respectively. Next, the Golgi segment was subtracted from the mitochondrial segment. The mean CIMPR fluorescence intensity was then determined in the latter mitochondrial-Golgi segment as an arbitrary but relative value. Background staining levels were obtained by quantifying mean signal levels on a whole cell segment subtracting the Golgi segment. Three biological repeats of this experiment were performed, measuring a total of ≥50 cells per cell line.

### Cell fractionation

For the cell fractionation experiment shown in Supplementary Figure 8, the protocol of Itzhak et al.^34^ was adapted. All steps were performed on ice with pre-chilled ice-cold buffers. A 9 cm dish of confluent cells for each cell line was washed once with PBS and incubated in PBS for 5 min. The cells were washed once with hypotonic lysis buffer (25 mM Tris-HCl, pH 7.5, 50 mM sucrose, 0.5 mM MgCl_2_ and 0.2 mM EGTA) prior to 5 min incubation in the same buffer. Next, the cells were scraped in 1 ml of fresh hypotonic lysis buffer and were transferred to a Dounce homogeniser for homogenisation with 50 strokes with the tight pestle. Following homogenisation, the sucrose concentration was immediately restored to 250 mM with hypertonic sucrose buffer (2.5 M sucrose, 25 mM Tris (pH 7.5), 0.5 mM MgCl_2_ and 0.2 mM EGTA) and the cell homogenates were spun at 10 000 × *g* for 10 min at 4 °C. The post-nuclear supernatant was then spun at 80 000 × *g* for 30 min in an Optima MAX-XP ultracentrifuge (Beckman Coulter). The pellets were resuspended in 1 ml SDS buffer made up in hypotonic buffer and SDS was added to the supernatant to a final concentration of 2.5%. All samples were heated for 5 min at 72 °C prior to addition of NuPAGE LDS Sample Buffer and boiling for 5 min. Equal volumes of all samples were loaded for SDS-polyacrylamide gel electrophoresis (SDS-PAGE). Three biological repeats of the experiment were performed.

### Western blotting

Cells were lysed in SDS buffer (2.5% SDS and 50 mM Tris, pH 8.0). Lysates were incubated at 65 °C, passed through a QIAshredder column (Qiagen) and boiled in NuPAGE LDS Sample Buffer for 3 min. Samples were loaded at equal protein amounts (or equal volumes for the fractionation experiments) for SDS-PAGE, performed on NuPAGE 4–12% Bis–Tris gels in NuPAGE MOPS SDS Running Buffer (Life Technologies). PageRuler Plus Prestained Protein Ladder (Thermo Fisher Scientific) was used to estimate the molecular size of bands. Proteins were transferred to nitrocellulose membrane by wet transfer and membranes were blocked in 5% w/v milk in PBS with 0.1% (v/v) Tween-20 (PBS-T). Primary antibodies (diluted in 5% milk) were added for at least 1 h at room temperature, followed by washing in PBS-T, incubation in secondary antibody (also in 5% milk) for 30 min at room temperature, washing in PBS-T and finally PBS. Chemiluminescence detection of horseradish peroxidase-conjugated secondary antibody/protein-A was carried out using AmershamECL Prime Western Blotting Detection Reagent (GEHealthcare) and X-ray film (Kodak). Where representative blots are shown, the experiment was repeated at least two times.

### Native immunoprecipitations

Cells were rinsed twice with ice-cold PBS, scraped into 1 ml ice-cold PBS-Tx (PBS adjusted to 1% Triton (93443, Sigma-Aldrich) and extracted for 20 min rotating at 8 rpm at 4 °C. The lysate was centrifuged at 20 000 × *g* for 20 min at 4 °C to pellet cell debris and incubated with 50 µl PA Sepharose bead slurry (50% v/v in PBS) 8 rpm at 4 °C to remove any nonspecific binding to the beads. The beads were removed and the lysates were incubated with primary antibody for 3 h, rotating at 8 rpm at 4 °C. This was followed by incubation with 50 µl PA Sepharose bead slurry for 1 h to capture the protein-antibody complexes. The beads were then washed four times in PBS-Tx and twice in 1 ml PBS to remove detergent. The soft elution protocol was used to elute the precipitated proteins off the beads^35^. In short, the beads were resuspended in soft elution buffer (0.2% (w/v) SDS, 0.1% (v/v) Tween-20 and 50 mM Tris-HCl, pH 8.0) and incubated for 7 min at 25 °C, shaking at 1000 rpm. This step was repeated twice and each time the supernatant was collected after spinning and pooled together. The eluted proteins were precipitated with acetone at −20 °C overnight and collected by spinning at 10 000 × *g* for 10 min. The final pellet was resuspended in sample buffer and run on an SDS-polyacrylamide gel for immunoblotting or mass spectrometry analysis on a Q Exactive mass spectrometer (Thermo Fisher Scientific).

### BioID biotin-streptavidin pull downs

RPE cells stably expressing BirA*-tagged WDR11, FAM91A1 or C17orf75, and control RPE cell lines expressing GFP-BirA* or BirA* only (a kind gift from Folma Buss, CIMR) were cultured in media containing 50 µM biotin for 24 h. The cells were harvested by scraping into 5 ml ice-cold PBS on ice, pelleted (600 × *g*, 5 min) and washed twice in PBS. Lysis was performed in 1 ml RIPA buffer (Tris-buffered saline (TBS; 50 mM Tris-HCl, pH 7.4, 150 mM NaCl), 1% NP-40, 0.5% sodium deoxycholate, 1 mM EDTA, 0.1% SDS and protease inhibitors). DNA was sheared by passing the lysate through a QIAshredder column and the lysates were extracted for 10 min rotating at 8 rpm at 4 °C. Sonication was performed using 3 × 5 s bursts with 5 s rests in between (amplitude of 10 microns). The lysates were then spun at 15 000 × *g* at 4 °C for 15 min. Biotinylated proteins were affinity-purified using Pierce High Capacity Streptavidin Agarose (Thermo Fisher Scientific) by incubating with the lysates for 3 h at 8 rpm at 4 °C. The beads were then pelleted and washed three times in 1 ml RIPA buffer, twice with 1 ml TBS, twice in 1 ml then once in 200 µl 50 mM ammonium bicarbonate, pH 8. The proteins were then reduced in 10 mM dithiothreitol, alkylated with iodoacetamide (50–55 mM) and digested with trypsin (1 µg; Roche). The eluted proteins were collected, spun at 15 000 × *g* for 10 min at room temperature, spiked with 1 µl of 100% trifluoroacetic acid and dried in a vacuum centrifuge (Eppendorf). The final protein pellet was analysed on a Q Exactive mass spectrometer (Thermo Fisher Scientific). Relative label-free quantification was performed across four biological replicate experiments.

Statistical analysis was performed using the Perseus MaxQuant analysis package (http://www.perseus-framework.org/). The protein identification lists were filtered by removing matches to the reverse database, proteins only identified with modified peptides and common contaminants. Abundance ratios were then log_2_-transformed and the list was further filtered by removing proteins with fewer than two valid values in at least one group (control or WDR11 complex samples). Two-tailed *t*-tests were performed for all identified proteins, across all replicates versus the control data set. Volcano plot analysis was used to identify enriched proteins, with the criteria: FDR-adjusted *P* value ≤0.01 and log_2_ fold change ≥2.

### Comparative genomic analysis

Potential orthologues of each component of the WDR11 complex were identified from a broad, representative distribution of eukaryotic genomes^36^ using both Basic Local Alignment Search Tool (BLAST)- and iterative HMM (jackhmmer)-based approaches with the human sequences as the initial query. Where no orthologues were identified in specific taxa, further searches were carried out using candidate orthologues from phylogenetically closer taxa as queries. Orthology of the candidate sequences was assessed using the reciprocal BLAST method against the non-redundant protein database as well as reciprocal jackhmmer searches using the candidate sequence as the initial query. Sequences were considered orthologous if they retrieved query sequences with an *E* value at least two orders of magnitude smaller than the next best hit from the same taxa. The full taxonomic distribution of WDR11 complex components across all taxa searched can be found in Supplementary Figure 2.

### Statistics

Statistical analyses were performed using GraphPad Prism 7. Data were tested for significance by unpaired two-tailed *t*-tests when two samples were compared or by one-way ANOVAs followed by Bonferroni post hoc test when more than two samples were compared. P values of statistical significance are represented as ***P < 0.001, **P < 0.01, *P < 0.05, ns *P* > 0.05.

### Data availability

All data supporting this work are available on reasonable request to the corresponding author.

## Electronic supplementary material

Supplementary Information

Peer Review File

Description of Supplementary Files

Supplementary Data 1
