# Supplementary material for: The WDR11 complex facilitates the tethering of AP-1-derived vesicles
Source: Nat Commun. 2018 Feb 9;9:596. doi: 10.1038/s41467-018-02919-4 (PMC5807400; doi:10.1038/s41467-018-02919-4)
Supplement: Supplementary file 1 — Supplementary Information [file 41467_2018_2919_MOESM1_ESM.pdf]

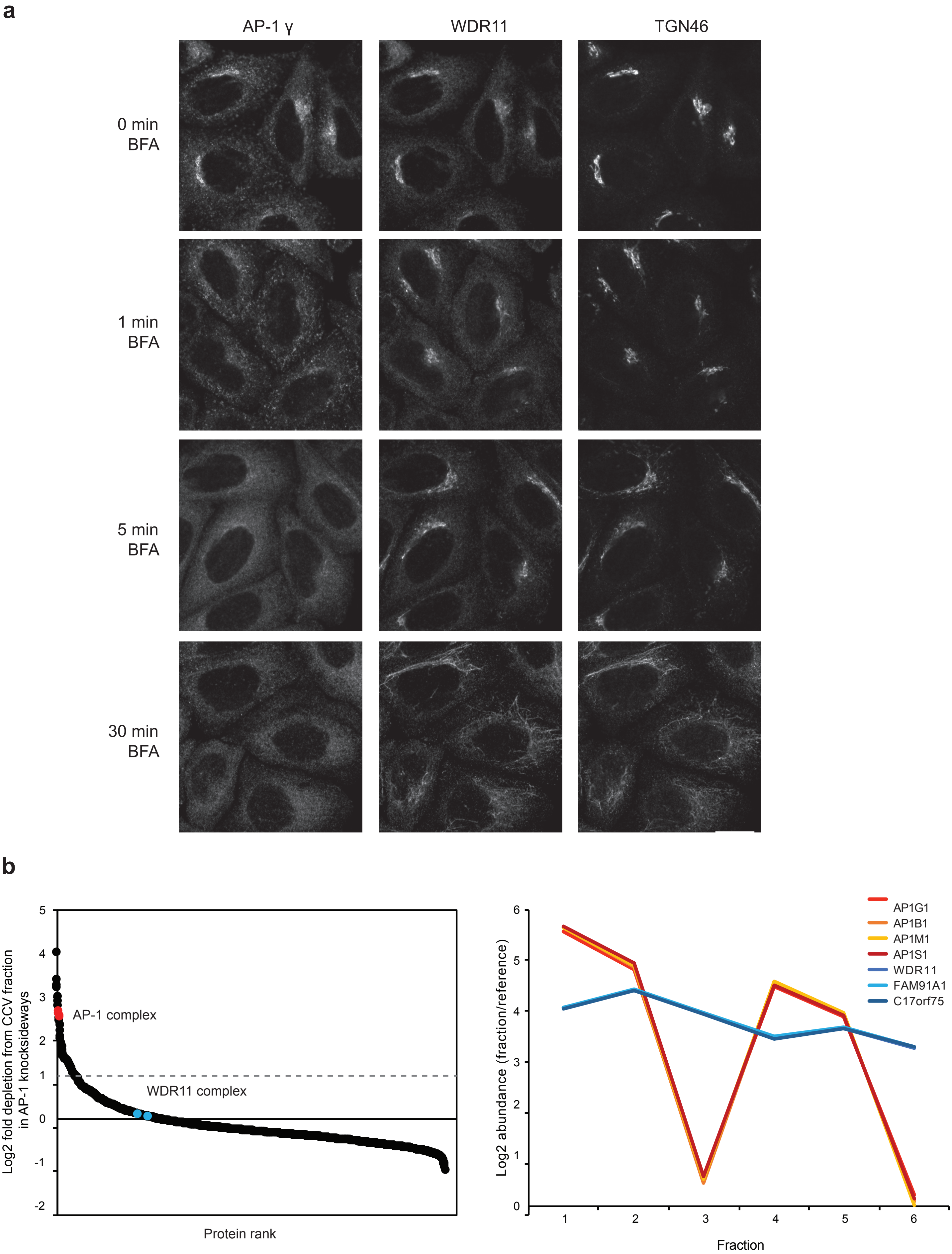

Supplementary Figure 1. Further evidence that the WDR11 and AP-1 complexes are not associated with each other. (a) Unlike AP-1, WDR11 is not BFA-sensitive. Cells were treated with 50 µg/ml BFA for the indicated lengths of time, then stained for WDR11, AP-1 γ, and TGN46. While BFA disrupted the TGN distribution of AP-1 γ after 1 min, WDR11 remained colocalised with TGN46, becoming tubular after 30 min. Scale bar: 20 µm. (b) The amount of WDR11 complex in a vesicle-enriched fraction is not affected by AP-1 knocksideways. Data were extracted from Hirst et al., 2012[1]; proteins more than 2-fold depleted (i.e., above the dotted line) are likely to be AP-1-dependent CCV proteins. (c) The WDR11 complex and AP-1 have very different fractionation profiles. Data were extracted from Borner et al., 2014[2].

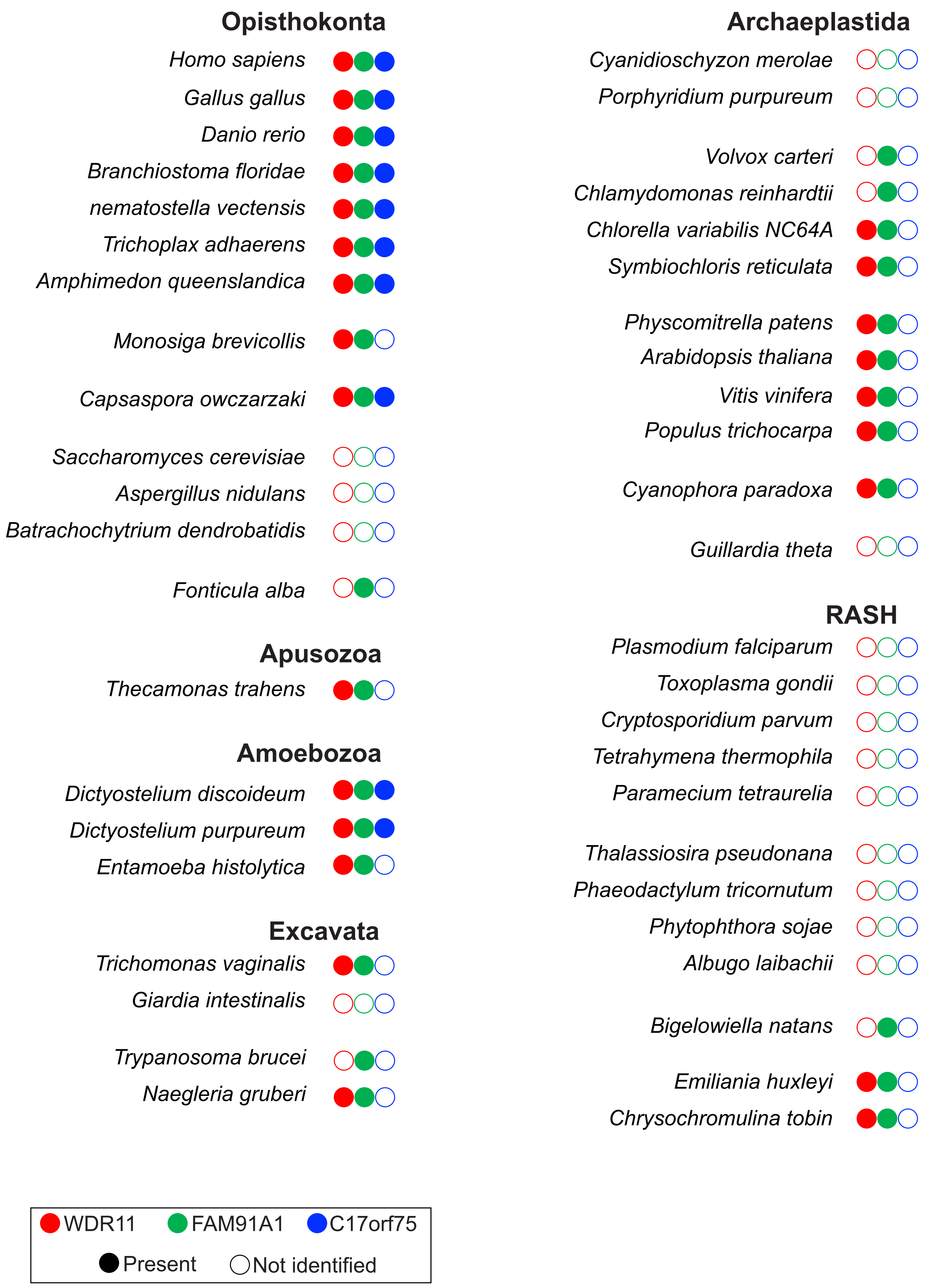

Supplementary Figure 2. Presence of WDR11, FAM91A1, and C17orf75 in diverse eukaryotes belonging to different supergroups. All of these organisms also have AP-1[3]. Acidic cluster-containing cargo proteins are found in many of them (e.g., XP_002677529 in Naegleria gruberi and XP_001033316 in Tetrahymena thermophila), suggesting that AP-1-dependent sorting of acidic cluster proteins may be an ancient mechanism, although there is no evidence that the WDR11 complex actually binds to acidic clusters.

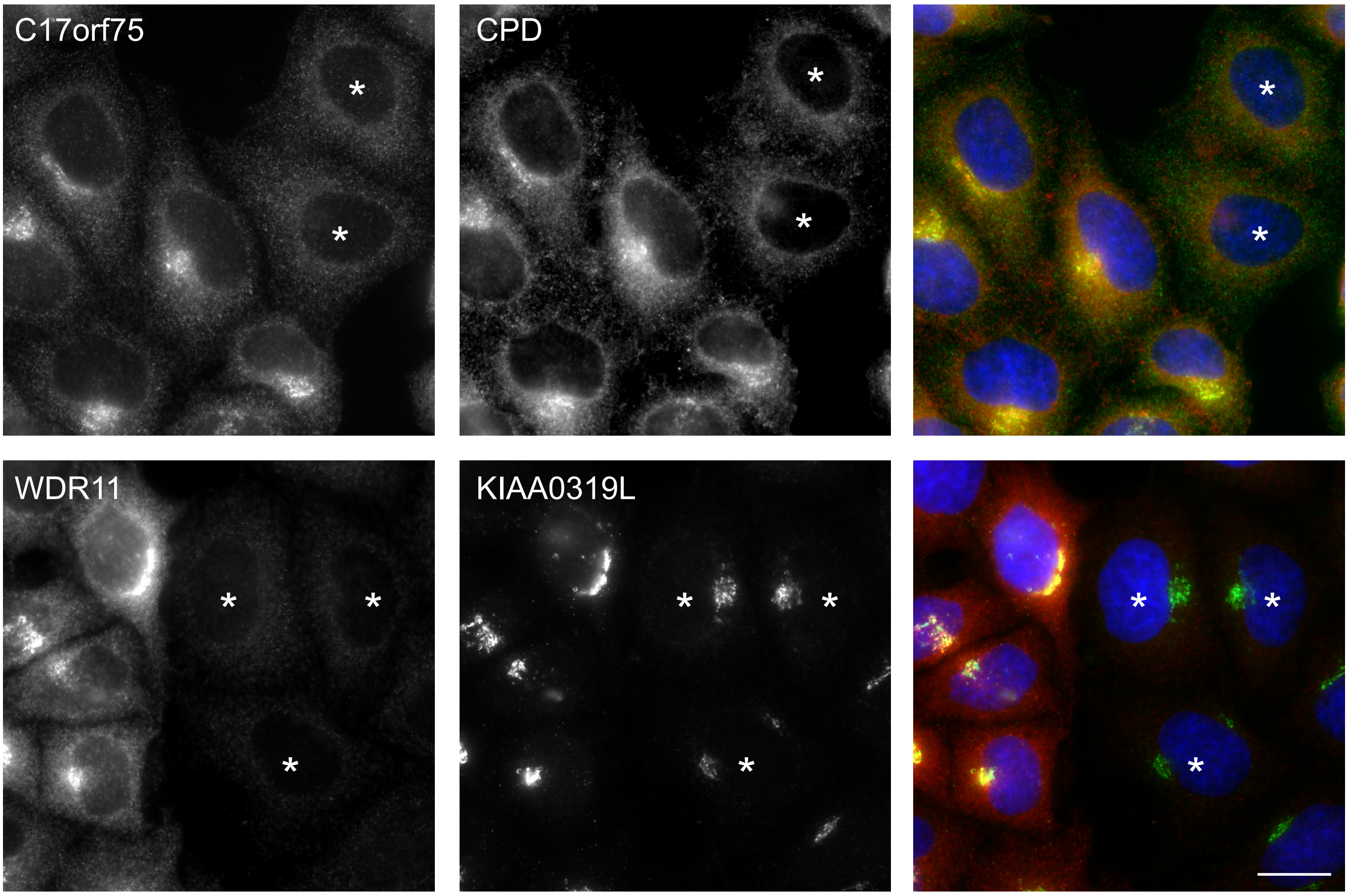

Supplementary Figure 3. AP-1 cargo localisation in WDR11 knockout cells. (a) Mixed populations of wild-type and WDR11-knockout cells were stained for the AP-1 cargo proteins CPD and KIAA0319L. Localisation of the cargo proteins is altered in the knockout cells (asterisks). CPD (red in the merged image), like CIMPR, appears more dispersed, although the pattern is difficult to discern because of background labelling by the antibody; while KIAA0319L (green in the merged image) becomes fainter, even though the total amount in the cell is the same (see Figure 1f). These different patterns may be due to differences in the interactions of the three proteins with other types of sorting machinery (e.g., AP complexes, GGAs, and retromer), which would affect their steady state distribution. Scale bar: 20 μm.

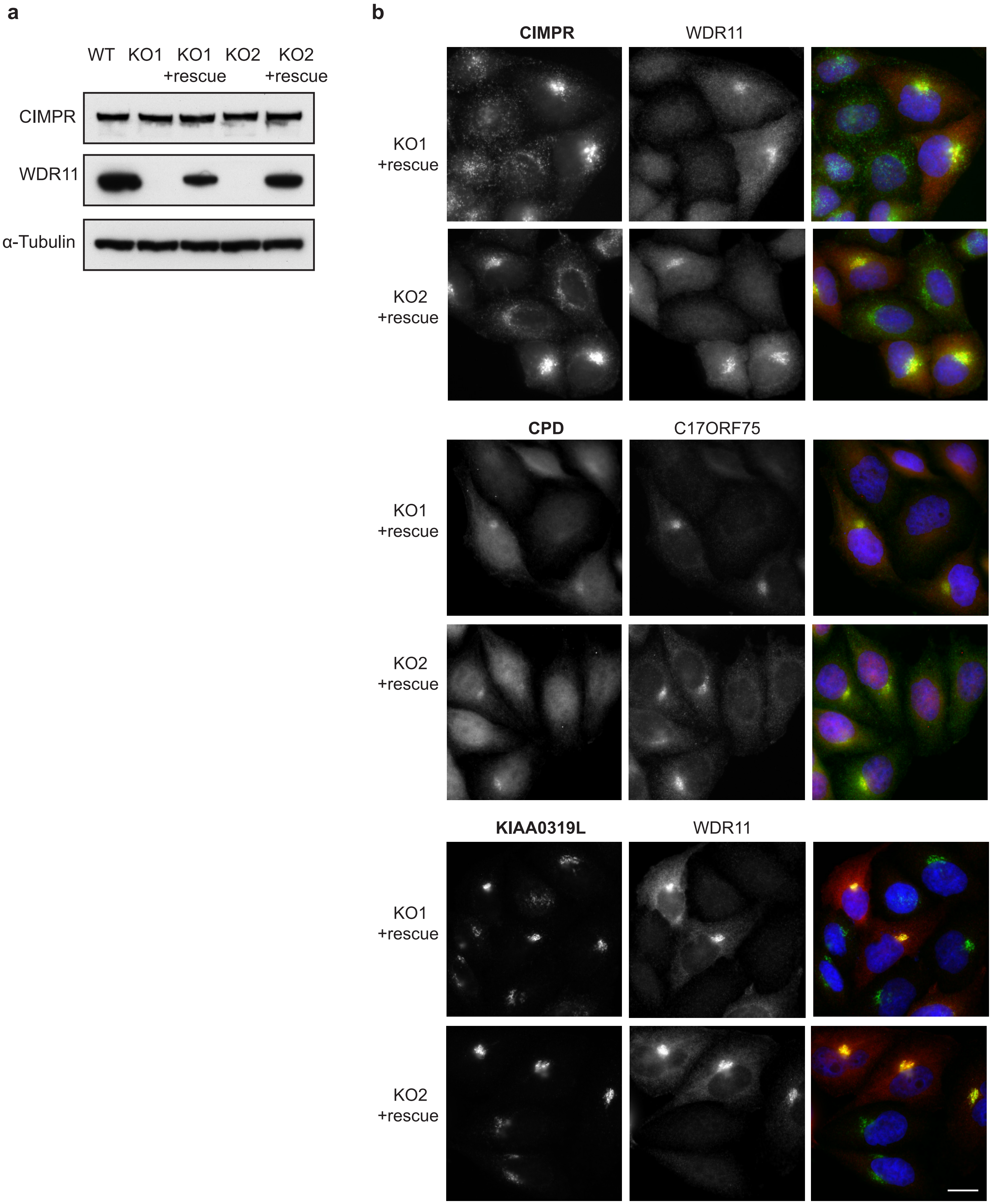

Supplementary Figure 4. Rescue of the cargo mislocalisation phenotype. (a) Western blot showing the rescue of both knockout cell lines with wild-type WDR11. (b) Immunofluorescence images of endogenous cargo proteins in mixed populations of knockout and rescued cells. In the merged images, CIMPR and KIAA0319L are green, while CPD is red. Scale bar: 20 μm.

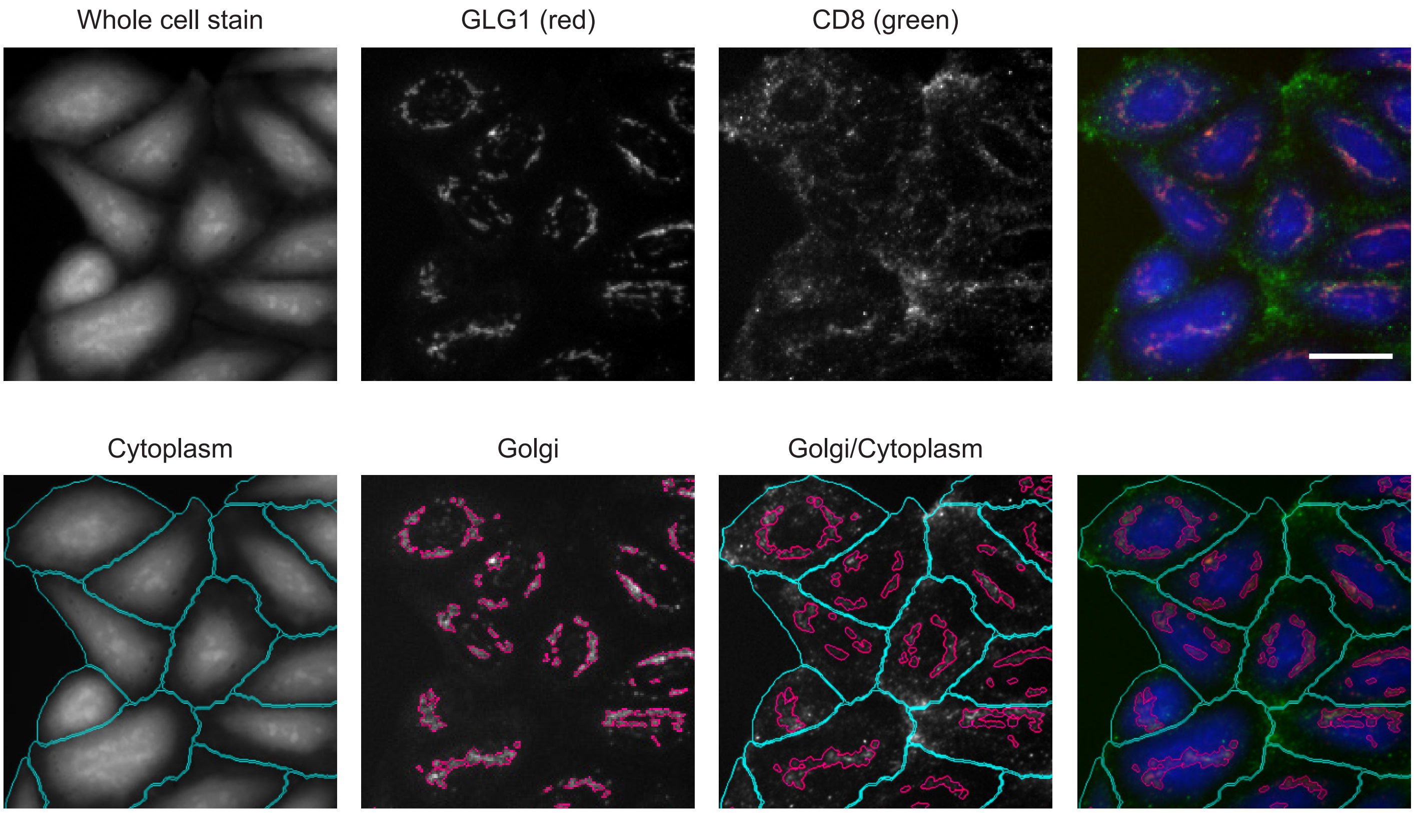

Supplementary Figure 5. Quantification of Golgi localisation of endocytosed antibody, using automated microscopy. A whole-cell stain was used to identify cell boundaries (cyan lines) and GLG1 labelling was used to delineate the Golgi region (pink lines). The CD8 fluorescence intensity was then measured in either the Golgi region or the rest of the cell (whole cell stain subtracting the Golgi region), and the Golgi-to-non-Golgi ratio was calculated. Scale bar: 20 µm.

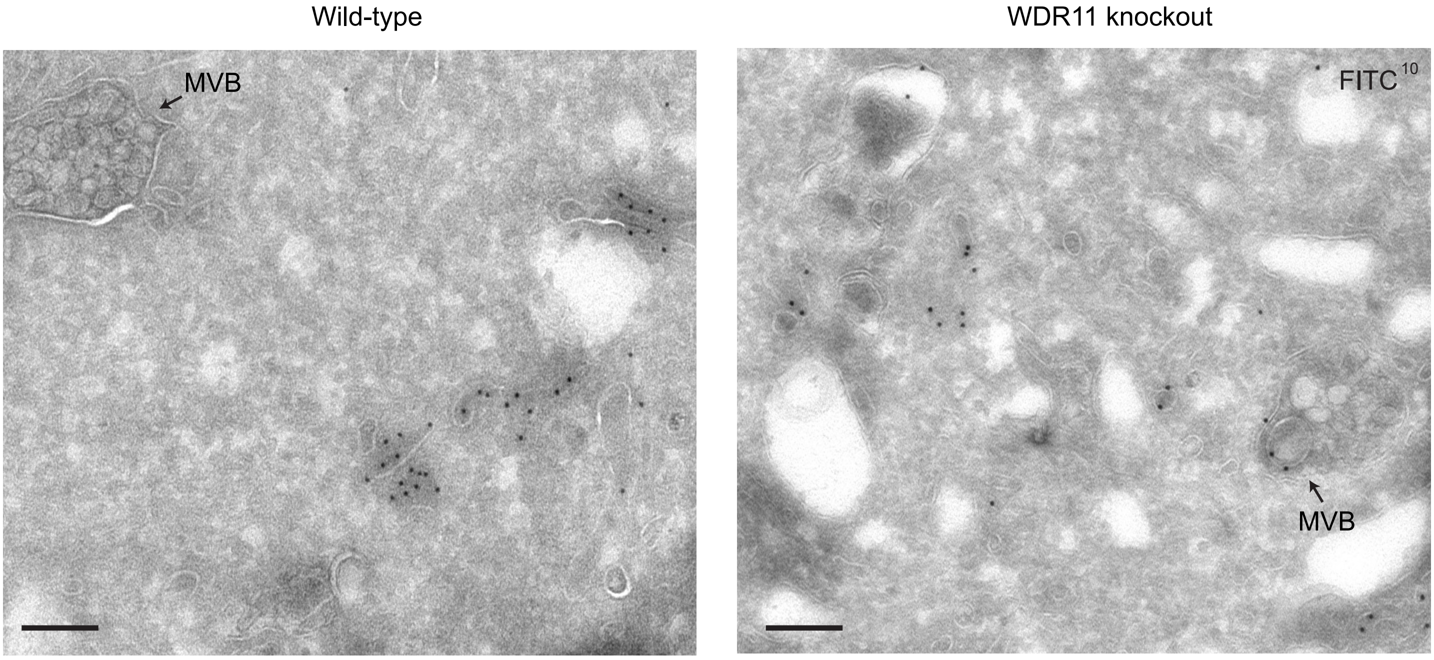

Supplementary Figure 6. Localisation of endocytosed anti-CD8-CIMPR. Wild-type and WDR11-knockout cells stably expressing CD8-CIMPR were allowed to endocytose FITC-conjugated anti-CD8 for three hours. The endocytosed antibody was localised on cryosections with anti-FITC followed by protein A coupled to colloidal gold. MVB: multivesicular body. Scale bars: 200 nm.

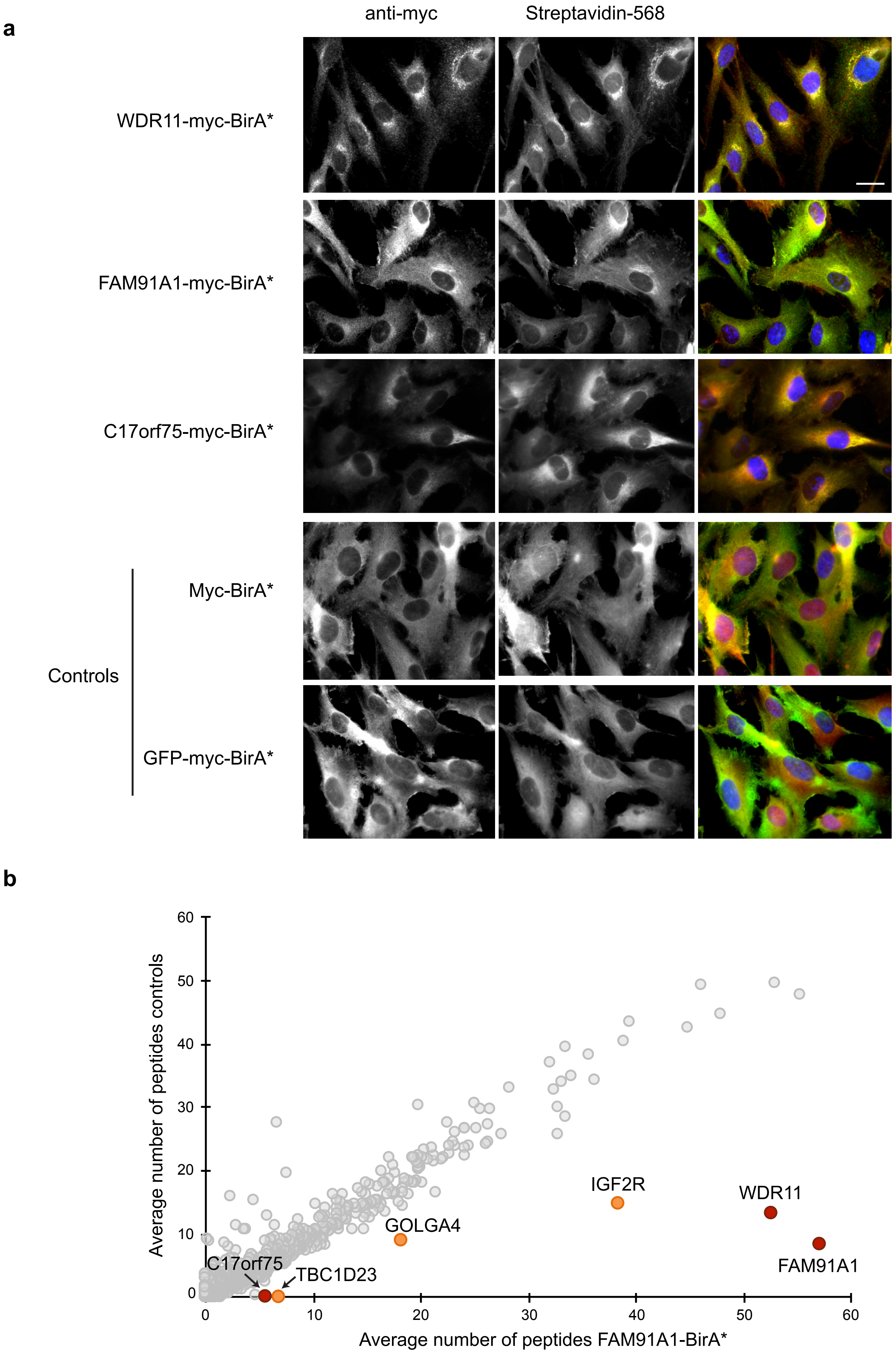

Supplementary Figure 7. Characterisation of retinal pigment epithelial (RPE) cell lines stably expressing constructs tagged with BirA* and myc. (a) The cells were grown in media containing biotin (50 µM) for 24 h and double labelled with streptavidin-568 (red) and anti-myc (green). The streptavidin and myc signals colocalise in the juxtanuclear region in cells expressing WDR11, FAM91A1, or C17orf75 constructs, but have a cytosolic distribution in control cell lines. Scale bar: 20 µm. (b) Peptide counts for biotinylated proteins in FAM91A1-myc-BirA* expressing cells were plotted against peptide counts for the same proteins in control cells. WDR11, FAM91A1, C17orf75, IGF2R (CIMPR), GOLGA4 (golgin-245), and TBC1D23 all show a clear enrichment in the FAM91A1-BirA* cells.

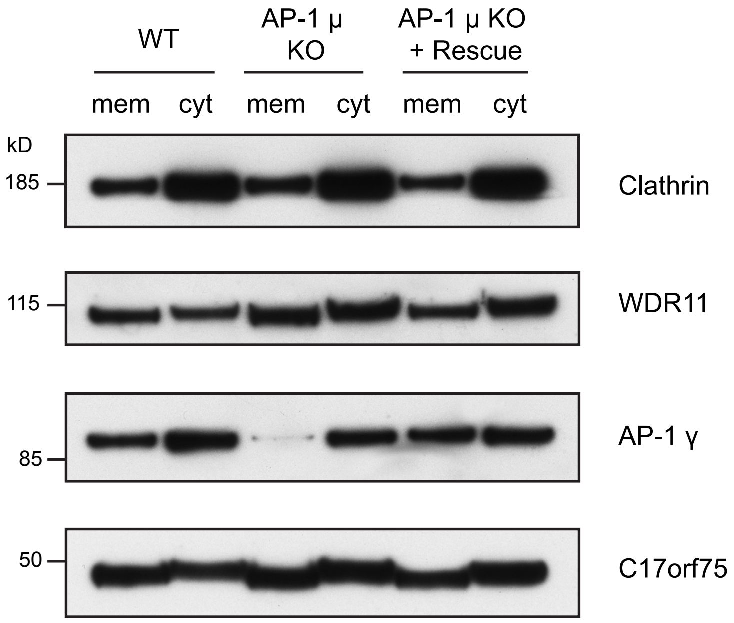

Supplementary Figure 8. Western blots of 80,000 x g supernatants and pellets from homogenates of wild-type, AP-1 µ1 knockout, and rescued AP-1 µ1 knockout cells. Both WDR11 and C17orf75 partition fairly equally between the membrane-containing pellet (mem = membranes) and the supernatant (cyt = cytosol). In contrast, there is almost no AP-1 γ in the pellet from the µ1 knockout cells, consistent with its cytosolic appearance by immunofluorescence (see Figure 7a).

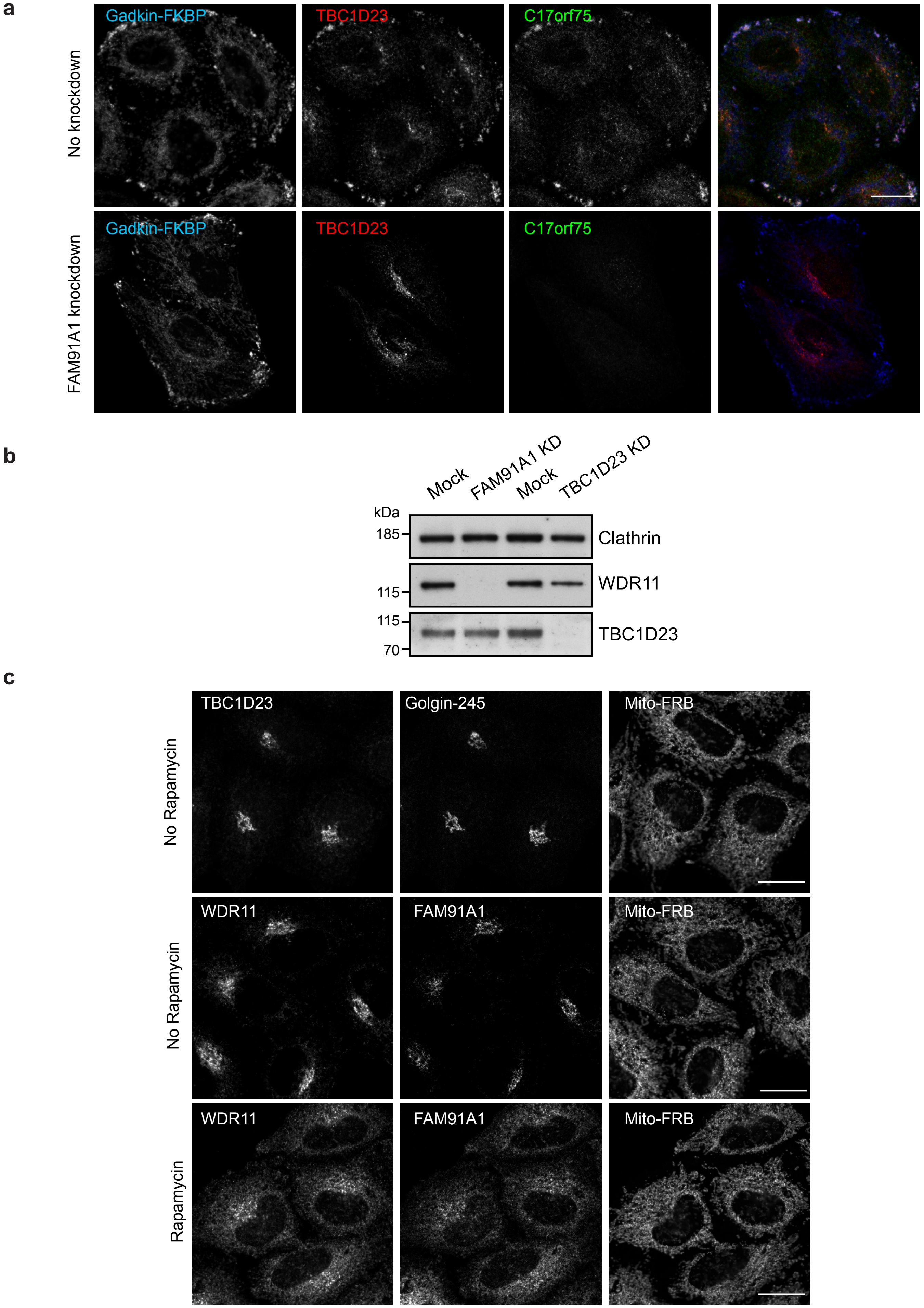

Supplementary Figure 9. Controls for the experiments shown in Figure 8. (a and b) Gadkin knocksideways cells were treated as in Figure 7d and then labelled for gadkin-FKBP, TBC1D23, and C17orf75, either with no knockdown (a) or after knocking down FAM91A1 (b). The three proteins colocalise in (a), while after the knockdown, TBC1D23 remains in the TGN region and C17orf75 is lost. (c) Western blot showing the efficiency of the knockdowns. (d) In the absence of rapamycin, TBC1D23, golgin-245, WDR11, and FKBP-tagged FAM91A1 all colocalise in the Golgi region in Mitotrap (Mito-FRB)-expressing cells. Addition of rapamycin causes the FAM91A1 to be rerouted to mitochondria. together with its partner WDR11, as well as TBC1D23 (see Fig. 8d). Scale bars: 20μm. Representative confocal images and Western blots are shown from three independent experiments.

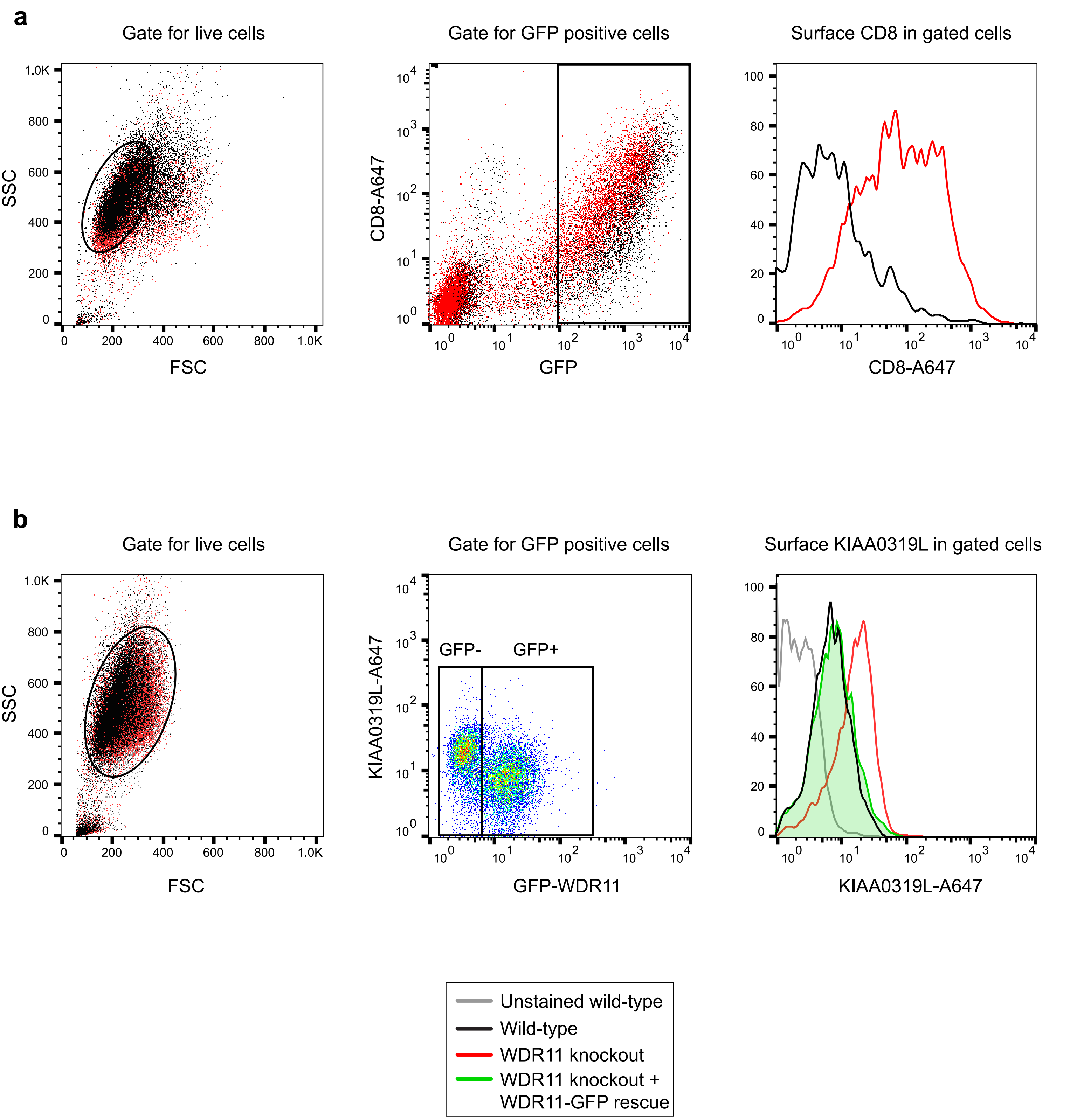

Supplementary Figure 10. FACS gating strategy for Figure 1 d and e. 10,000 live cells identified by their FSC/SSC profile (left panel) were analysed for each sample. (a) CD8 chimeras were co-transfected with an EGFP-N2 plasmid. GFP positive cells were gated (middle panel) to account for tranfetion efficiency. The mean surface CD8 fluorescence intensity was then calculated from this gated population (right). (b) In the WDR11-GFP rescue experiments, GFP positive and negative cells were gated (middle panel). The mean CIMPR or KIAA0319L fluorescence intensity was then calculated from the gated populations (right) and compared with that of wild-type and knockout cells.

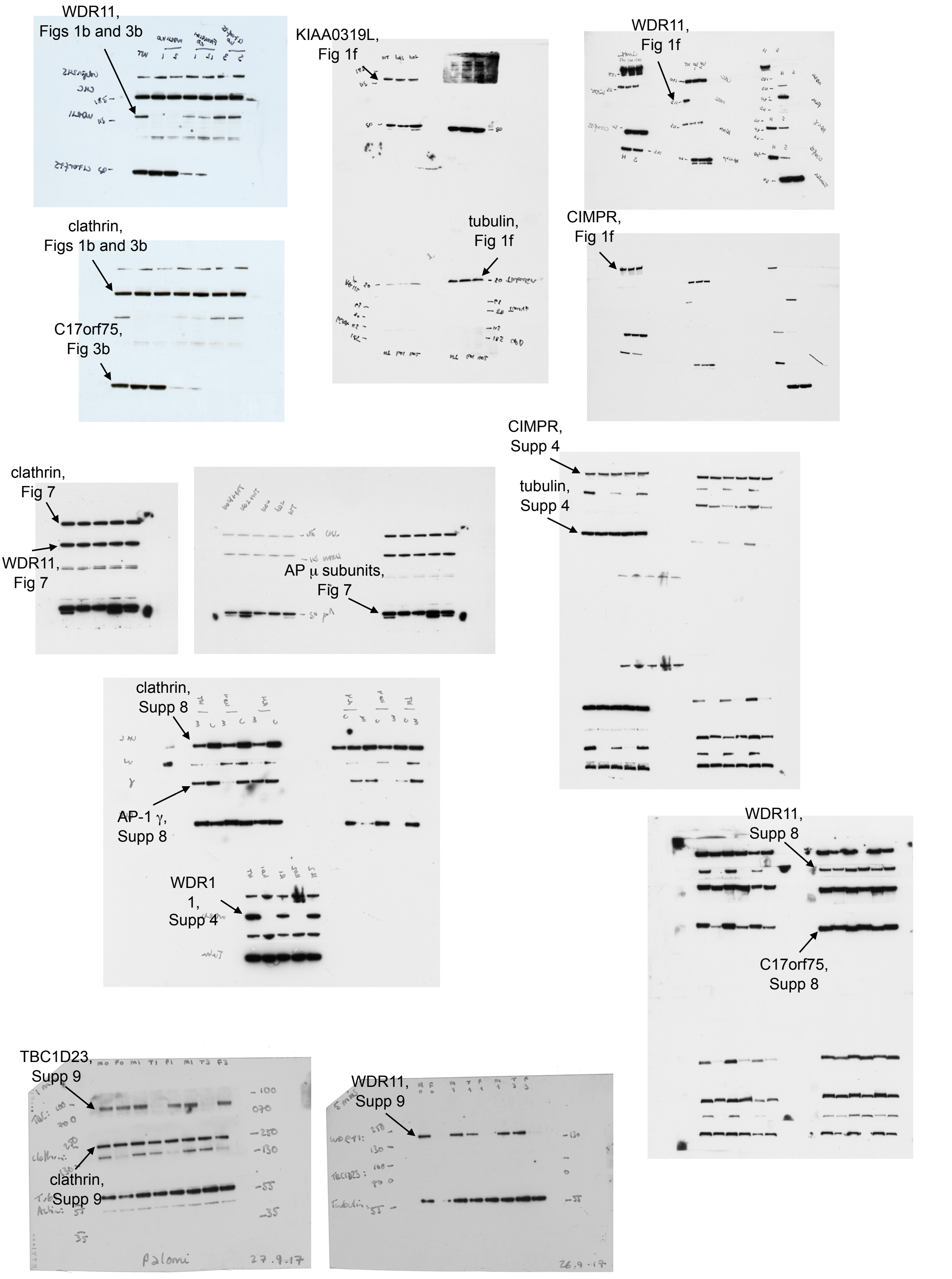

Supplementary Figure 11. Uncropped scans of all the Western blots. The relevant lanes and the figures in which they appear are indicated on each blot.

Protein names	Gene names	Unique peptides WT	Unique peptides KO
WD repeat-containing protein 11	WDR11	43	0
Protein FAM91A1	FAM91A1	32	0
Protein Njmu-R1	C17orf75	15	0
E3 ubiquitin-protein ligase TRIM21	TRIM21	12	11
Phospholipase DDHD1	DDHD1	8	0
Heat shock cognate 71 kDa protein	HSPA8	6	9
60S acidic ribosomal protein P0	RPLP0	6	1
78 kDa glucose-regulated protein	HSPA5	5	9
Endoplasmin	HSP90B1	4	7
Polyadenylate-binding protein 1	PABPC1	3	0
Protein-L-isoaspartate O-methyltransferase	PCMT1	3	1
Nucleolin	NCL	2	1
60S ribosomal protein L12	RPL12	2	0
La-related protein 1	LARP1	2	0
Uncharacterized family 31 glucosidase KIAA1161	KIAA1161	2	0
Ig gamma-3 chain C region	IGHG3	1	1
SUN domain-containing protein 5	SUN5	1	0
POU domain, class 3, transcription factor 4	POU3F4	1	1
Centrobin	CNTROB	1	0
Differentially expressed in FDCP 6 homolog	DEF6	1	0
Tyrosyl-DNA phosphodiesterase 2	TDP2	1	0
Sorting nexin-10	SNX10	1	0
60S ribosomal protein L11	RPL11	1	0
Zinc finger and BTB domain-containing protein 2	ZBTB2	1	3
Transcriptional activator protein Pur-alpha	PURA	1	0
Heterogeneous nuclear ribonucleoprotein H	HNRNPH1	1	1
Polyubiquitin-C	UBC	1	0
C-type lectin domain family 4 member F	CLEC4F	1	0
Heterogeneous nuclear ribonucleoprotein U	HNRNPU	1	0
40S ribosomal protein SA	RPSAP58	1	0
Glyceraldehyde-3-phosphate dehydrogenase	GAPDH	1	1
Stress-70 protein, mitochondrial	HSPA9	1	2
F-box/WD repeat-containing protein 1A	BTRC	1	0
Methylcytosine dioxygenase TET2	TET2	0	2
Myosin-10	MYH10	0	2
Junction plakoglobin	JUP	0	2
Desmoplakin	DSP	0	4
Heat shock 70 kDa protein 6	HSPA6	0	2
Glycine--tRNA ligase	GARS	0	3
Dermcidin	DCD	0	1
Calmodulin-like protein 5	CALML5	0	1
Zinc finger protein 639	ZNF639	0	3

Supplementary Table 1. Proteins identified in immunoprecipitates using anti-WDR11 from wild-type and WDR11 knockout cells. The proteins are ranked by number of peptides identified in wild-type cells. The top hits, WDR11, FAM91A1, and C17orf75, all come down in the wild-type cells only. Proteins detected in both immunoprecipitates are non-specific. The significance of DDHD1, a phospholipase, is unclear, but it was not detected as a hit by fractionation profiling, and fewer peptides were identified relative to its size than for the three established subunits of the complex.

SUPPLEMENTARY REFERENCES

1. Hirst, J. *et al.* Distinct and overlapping roles for AP-1 and GGAs revealed by the "knocksideways" system. *Curr. Biol.* **22**, 1711-1716 (2012).
2. Borner, G.H.H. *et al.* Fractionation profiling: a fast and versatile approach for mapping vesicle proteomes and protein-protein interactions. *Mol. Biol. Cell* **15**, 3178-3194 (2014).
3. Robinson, M.S. Forty years of clathrin-coated vesicles. *Traffic* **16**, 1210-1238 (2015).
