# Supplementary material for: The WDR11 complex facilitates the tethering of AP-1-derived vesicles
Source: Nat Commun. 2018 Feb 9;9:596. doi: 10.1038/s41467-018-02919-4 (PMC5807400; doi:10.1038/s41467-018-02919-4)
Supplement: Supplementary file 3 — Description of Supplementary Files [file 41467_2018_2919_MOESM3_ESM.pdf]

Description of Supplementary Files

File Name: Supplementary Data 1
Description: **Proteins enriched in the WDR11, FAM91A1 and C17orf75 BioID datasets.** The log2 fold change relative to the control dataset is given. Proteins highlighted in volcano plots have been similarly highlighted in the table.
