# Supplementary material for: The WDR11 complex facilitates the tethering of AP-1-derived vesicles
Source: Nat Commun. 2018 Feb 9;9:596. doi: 10.1038/s41467-018-02919-4 (PMC5807400; doi:10.1038/s41467-018-02919-4)
Supplement: Supplementary file 2 — Peer Review File [file 41467_2018_2919_MOESM2_ESM.pdf]

Editorial Note: this manuscript has been previously reviewed at another journal that is not operating a transparent peer review scheme. This document only contains reviewer comments and rebuttal letters for versions considered at Nature Communications.
